# Characterization of *α*-Glucosidase Inhibitors from *Psychotria malayana* Jack Leaves Extract Using LC-MS-Based Multivariate Data Analysis and In-Silico Molecular Docking

**DOI:** 10.3390/molecules25245885

**Published:** 2020-12-12

**Authors:** Tanzina Sharmin Nipun, Alfi Khatib, Zalikha Ibrahim, Qamar Uddin Ahmed, Irna Elina Redzwan, Mohd Zuwairi Saiman, Farahaniza Supandi, Riesta Primaharinastiti, Hesham R. El-Seedi

**Affiliations:** 1 Pharmacognosy Research Group, Department of Pharmaceutical Chemistry, Kulliyyah of Pharmacy, International Islamic University Malaysia, Kuantan 25200, Malaysia; tsn.np99@gmail.com (T.S.N.); zalikha@iium.edu.my (Z.I.); quahmed@iium.edu.my (Q.U.A.); elina@iium.edu.my (I.E.R.); 2 Department of Pharmacy, Faculty of Biological Sciences, University of Chittagong, Chittagong 4331, Bangladesh; 3 Faculty of Pharmacy, Airlangga University, Surabaya 60155, Indonesia; r_nastiti@gmail.com; 4 Institute of Biological Sciences, Faculty of Science, University of Malaya, Kuala Lumpur 50603, Malaysia; farahaniza@um.edu.my; 5 Center for Research in Biotechnology for Agriculture (CEBAR), Faculty of Science, University of Malaya, Kuala Lumpur 50603, Malaysia; 6 Pharmacognosy Group, Department of Pharmaceutical Biosciences, BMC, Uppsala University, SE-751 23 Uppsala, Sweden; hesham.el-seedi@farmbio.uu.se; 7 International Research Center for Food Nutrition and Safety, Jiangsu University, Zhenjiang 212013, China

**Keywords:** *Psychotria malayana* Jack, type 2 diabetes, *α*-glucosidase inhibitors, LC-MS, metabolomics, molecular docking

## Abstract

*Psychotria malayana* Jack has traditionally been used to treat diabetes. Despite its potential, the scientific proof in relation to this plant is still lacking. Thus, the present study aimed to investigate the *α*-glucosidase inhibitors in *P.* *malayana* leaf extracts using a metabolomics approach and to elucidate the ligand–protein interactions through in silico techniques. The plant leaves were extracted with methanol and water at five various ratios (100, 75, 50, 25 and 0% *v/v*; water–methanol). Each extract was tested for *α*-glucosidase inhibition, followed by analysis using liquid chromatography tandem to mass spectrometry. The data were further subjected to multivariate data analysis by means of an orthogonal partial least square in order to correlate the chemical profile and the bioactivity. The loading plots revealed that the *m/z* signals correspond to the activity of *α*-glucosidase inhibitors, which led to the identification of three putative bioactive compounds, namely 5′-hydroxymethyl-1′-(1, 2, 3, 9-tetrahydro-pyrrolo (2, 1-*b*) quinazolin-1-yl)-heptan-1′-one (**1**), *α*-terpinyl-*β*-glucoside (**2**), and machaeridiol-A (**3**). Molecular docking of the identified inhibitors was performed using Auto Dock Vina software against the crystal structure of *Saccharomyces cerevisiae* isomaltase (Protein Data Bank code: 3A4A). Four hydrogen bonds were detected in the docked complex, involving several residues, namely ASP352, ARG213, ARG442, GLU277, GLN279, HIE280, and GLU411. Compound **1**, **2**, and **3** showed binding affinity values of −8.3, −7.6, and −10.0 kcal/mol, respectively, which indicate the good binding ability of the compounds towards the enzyme when compared to that of quercetin, a known *α*-glucosidase inhibitor. The three identified compounds that showed potential binding affinity towards the enzymatic protein in molecular docking interactions could be the bioactive compounds associated with the traditional use of this plant.

## 1. Introduction

Diabetes mellitus (DM) is the most common chronic and metabolic disease, which affects a large number of populations in the world. Currently, more than 150 million people are suffering from diabetes, and this number is expected to reach 300 million by 2025 [1]. It is characterized by an elevated blood glucose level due to defects in insulin secretion and insulin resistance, or both. DM is associated with eye, renal, cardiovascular, and neurological complications in the long term and is also associated with symptoms such as polyuria, fatigue, weight loss, delayed wound healing, blurred vision, increases in plasma and urine glucose levels [2,3,4]. Generally, DM can be categorized into two types; type 1 and type 2. Many synthetic medicines like insulin and oral hypoglycemic drugs available on the market are used to treat type 2 diabetes, but the long term use of insulin therapy and oral synthetic medicines may cause severe side effects [5]. Furthermore, oral anti-diabetic drugs are costly; hence, it is very difficult for low and middle-income people to continually use these medications [6]. Taking into account the side effects of synthetic anti-diabetic drugs, the interest in herbal medicinal products for DM treatment is growing [7]. Several phytochemical constituents, including flavonoids, phenolic, coumarins, and other compounds that are plentiful in medicinal herbs have been evaluated as being capable of lowering blood glucose levels [8]. The capability of medicinal plants in treating DM relates to their ability to improve the action of pancreatic tissue by increasing insulin secretion or reducing intestinal glucose absorption [9]. Decreasing postprandial hyperglycemia is a therapeutic approach used for the treatment of DM. This goal can be achieved by inhibiting the carbohydrate hydrolyzing enzyme, *α*-glucosidase (AG). The AG enzyme plays a vital role in carbohydrate metabolism by breaking starch and disaccharides into glucose [10]. Therefore, inhibition of AG is an effective therapeutic approach for the management of DM.

*Psychotria malayana* Jack plant, which belongs to the Rubiaceae family, is widespread throughout tropical and subtropical countries. This plant is largely distributed in the west Indonesian archipelago and known to Lombok people as “lolon jarum” [11]. Lombok people traditionally used this plant for the treatment of wounds, skin infections and other skin diseases [11]. People in India, Indonesia and Brazil, have conventionally used this plant to treat digestive problems, stomach pain and infections of the female reproductive system [12]. Karo people in North Sumatra used the species of *Psychotria*, which is locally known as “loning” for the treatment of diabetes [13]. Khaled et al. [12] showed the anti-diabetic property of this plant on diabetic-induced zebrafish. Although several alkaloids in this plant have been reported, none of these alkaloids have been found to exert anti-diabetic activity. More research should be performed to identify bioactive compounds related to the anti-diabetic properties of this plant.

LC-MS-based metabolomics has been used to identify bioactive compounds in medicinal herbs [14]. Condensed tannins and flavonoid derivatives have been identified as antioxidant metabolites from the leaves of *Fragaria vesca* by using LC-MS based metabolomics [15]. Furthermore, Kamalrul et al. [16] identified eight compounds from *Wedelia trilobata* leaves that exhibited potential allelopathic effects. Liquid chromatography (LC) combined with quadrupole time-of-flight (Q-TOF) mass spectrometry (MS) was used to annotate metabolites that could act as *α*-glucosidase (AG) inhibitors [17]. Suganya et al. [17] reported AG inhibitors from *Clinacanthus nutans* leaves using a similar technology. Additionally, Gilda et al. [18] identified 35 metabolites, along with antioxidant and AG inhibitory effects of the fruits of both *Morus alba* and *Morus nigra*. Similar technology has been applied to evaluate the activity of *Clinacanthus nutans* leaves against cisplatin toxicity [19]. In addition, potential thrombin/factor Xa inhibitors were reported from *Salvia miltiorrhiza* Bunge and *Ligusticum chuanxiong* Hort using the LC-MS-based multivariate data analysis technique [20,21]. Chromatographic technique-based metabolomics was also applied to compare and reveal the chemical profiles of raw and fermented Chinese Ge-Gen-Qin-Lian decoction (a traditional chinese medicine formula) during investigation of its anti-diabetic effects on high-fat diet and streptozotocin-induced rats [22]. The choice of using LC-MS is due to the technique having a high mass resolution and detection sensitivity in regard to mass detection, thus, it can precisely measure the mass [23,24]. LC-Q-TOF-MS provides improved detection for fragmented ions with a high resolution and mass precision in comparison to many other tandem mass spectrometries [23].

Molecular docking plays an important role in predicting the binding modes and binding abilities of small molecules towards the targeted proteins, which is crucial in designing potential drugs [25]. In the present study, the activity of *P. malayana* Jack leaf extracts were investigated through an AG inhibition assay. Subsequently, the AG inhibitors in this herb were identified using LC-MS-based metabolomics. An in silico study was carried out to predict the molecular interaction between the AG enzyme (protein) and the tentative AG inhibitors (ligands) that were identified in *P. malayana* leaf extracts.

## 2. Results

### 2.1. α-Glucosidase Inhibition (AGI) of the Plant Extract

**Figure 1:**
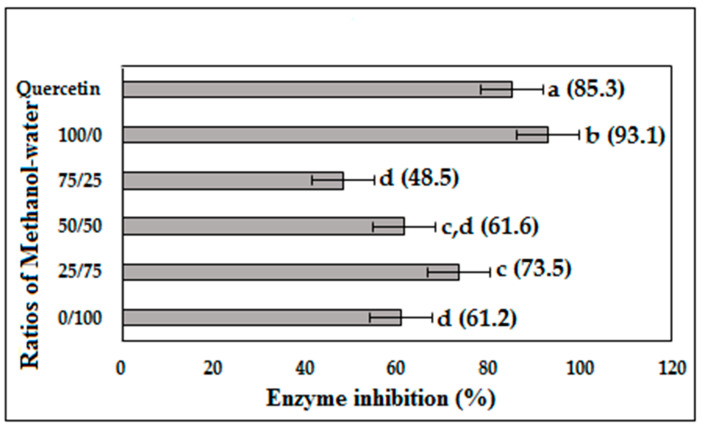
Percentage of *α*-glucosidase inhibition at the concentration of 10 µg/mL of *P. malayana* leaves extracts. The values that do not share the same letter are significantly different (*p* < 0.05), as measured by Tukey’s comparison test.

Figure 1 shows the *α*-glucosidase inhibition (AGI) activity of five extracts of *P. malayana* leaves at the assay concentration of 10 µg/mL. The highest AGI activity (93.1% inhibition) was shown by 100% methanol, followed by the 25% methanol extract with an AGI activity of 73.5% inhibition. However, the AGI activity of the 25% methanol was not statistically significant (*p* < 0.05) from 50% methanol (61.6% inhibition). The AGI activity of 50% methanol and water extract (61.2% inhibition) was not significantly different (*p* < 0.05), but was lower than that of the 25% methanol extract. The lowest AGI activity was shown by the 75% methanol extract (48.5% inhibition). The AGI activity of the different *P. malayana* leaf extracts exhibited the following trend: 100 > 25 > 50 > 0 > 75% *v/v* methanol extract.

### 2.2. Multivariate Data Analysis

Supervised multivariate data analysis, by means of orthogonal partial least square (OPLS), was used to correlate the x variables (LC-MS signals) and the y variable (AGI activity) of each sample. The mass to charge ratio (*m/z*) and retention time were considered as the x variables and resulted from the pre-processing of LCMS data using an online Mzmine platform. Pareto scaling was used in order to suppress the effect of *m/z* noise in the statistical calculation, thus avoiding a biased result.

**Figure 2:**
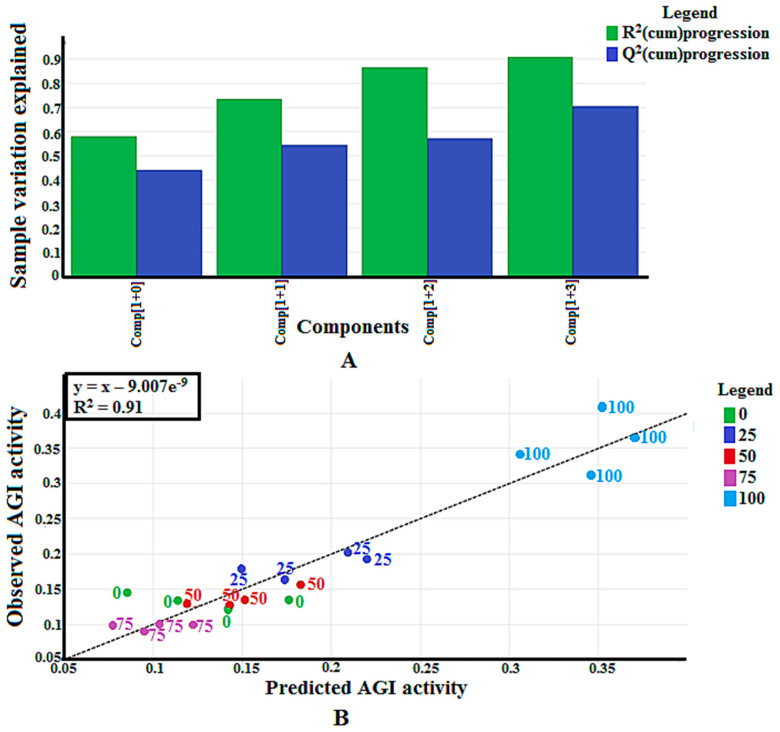
(**A**) Summary of fit of established orthogonal partial least square (OPLS) model for *P. malayana* leaf extracts; (**B**) Observed vs. predicted *α*-glucosidase inhibition (AGI) activity with R^2^ value of 0.91 from 20 extracts of *P. malayana* leaves.

The Y-axis in Figure 2A represents the variation explained by the calibration model, while the R^2^(cum)progression is the degree of variation that can be explained by each of the components. The variation was calculated as a cumulative score and thus, described in a progression manner. The OPLS model produced four principal components (PC), explaining 91% of the total variation (see Figure 2A). PC1 explained the largest variation of the samples (58%), while PC2, PC3, and PC4 represented 16%, 29%, and 33% of the sample variation, respectively. The difference between the R^2^Y and Q^2^Y of all the PCs in this study was not larger than 0.3, confirming the validation of this multivariable calibration model, according to Eriksson et al. [26]. Another validity evaluation of this model was performed through the analysis of the regression coefficient (R^2^) between the predicted and actual AGI activities of the samples (Figure 2B). The R^2^ value obtained was 0.91, indicating the validity of this model since it was higher than 0.90 [26]. The permutation test showed that the R^2^Y and Q^2^Y intercept values of this model were lower than 0.4 and 0.05, respectively, confirming that this calibration model was valid [26].

**Figure 3:**
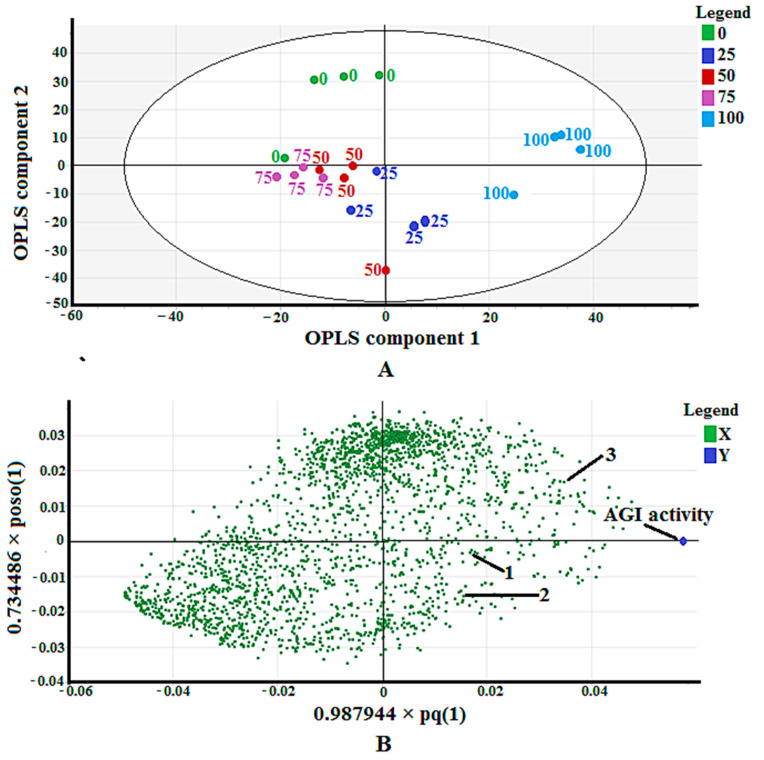
(**A**) Score scatter plot of validated OPLS model for 20 extracts of *P. malayana* leaves; (**B**) The loading scatter plot of OPLS model.

Figure 3A shows a score scatter plot that displays the separation among the samples. The sample separation can be viewed well through a combination of OPLS component one and two. The most active extract (100% methanol) was separated in the positive side, while the others—except part of the 25% methanol extract—were separated in the negative side of OPLS component one. While OPLS component two could not separate the most active extract from the less active extracts.

The loading scatter plot, as shown in Figure 3B, maps out the correlation of the x variables (*m/z*) to the y variable (AGI activity). The *m/z* values being closer to the AGI activity indicates their positive correlation to the bioactivity. Plenty of *m/z* vindicated this property. However, only three of them (*m/z* of 315.2, 317.2, and 349.2) could be identified after comparison with the available databases and publications. The detailed explanation of compound identification is described in Section 2.3. The three identified putative compounds are (5′-hydroxymethyl-1′-(1, 2, 3, 9-tetrahydro-pyrrolo [2, 1-*b*] quinazolin-1-yl)-heptan-1-one) (**1**), *α*-terpinyl-*β*-glucoside (**2**), and machaeridiol-A (**3**).

### 2.3. Identification of Putative Compounds

**Figure 4:**
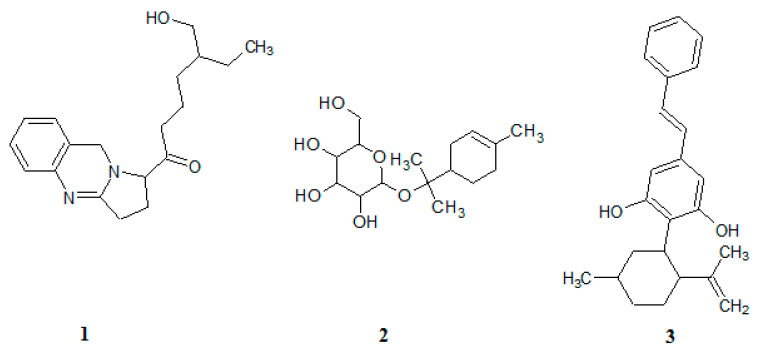
Chemical structures of compound **1**, **2**, and **3**.

Identification of putative compounds was performed by analyzing the *m/z* spectrum of fragmented ions of each compound which was obtained from the LC-MS/MS instrument with a positive ionization mode (see Table 1). The fragmentation pathway of three compounds is shown in Figure S1, the supplementary file. The chemical structures of the identified compounds are shown in Figure 4. 

Compound **1,** an alkaloid, consists of a quinazoline ring with an aliphatic side chain containing carbonyl and hydroxyl groups. Compound **1** was protonated at the oxygen attached to the O-H functional groups to form the parent ion [M + H]^+^ having an *m/z* of 315. Subsequent removal of C_2_H_5_, C_4_H_3_, C_4_H_10_, C_4_H_10_O, C_11_H_19_O, C_13_H_20_N_2_O_2_ and C_17_H_24_N_2_O_2_ from the parent ion resulted in ions with *m/z* values of 286, 264, 257, 241, 132, 79, and 27, respectively. Removal of a methyl group from the parent ion produced an ion with an *m/z* of 300. The loss of methane (CH_4_) from the parent ion results in the formation of an ion with an *m/z* of 299 from which dihydrogen was removed to produce an ion with an *m/z* of 297. Removal of CH_4_O from the parent ion produced an ion with an *m/z* of 283, and the further loss of hydrogen caused the formation of an ion with an *m/z* of 282. In another pathway, the parent ion lost pentanol (C_5_H_12_O) to form an ion with an *m/z* of 227, which was further fragmented to produce an ion with an *m/z* of 185 by removal of ethenone (C_2_H_2_O). The loss of heptanol (C_7_H_16_O) from the parent ion leads to the formation of an ion with an *m/z* of 199, from which, further loss of ethylene (C_2_H_4_) forms an ion with an *m/z* of 171. Further fragmentation of this ion leads to the formation of an ion with an *m/z* of 157 by removal of methylene (CH_2_). The parent ion lost benzene (C_6_H_4_) to produce an ion with an *m/z* of 239 that was further fragmented to produce an ion with an *m/z* of 139 by removal of hexanal (C_6_H_12_O). Later on, a water molecule was removed to produce an ion with an *m/z* of 121 that led to the production of another ion with an *m/z* of 93 by removal of ethylene (C_2_H_4_).

Compound **2** is a diterpenoid glucoside which was protonated at the oxygen atom of one of the hydroxyl groups of glycoside during positive Electrospray Ionisation (ESI) to form the parent ion [M + H]^+^ with an *m/z* of 317. The removal of C_4_H_6_, C_11_H_20_O_6_, C_14_H_24_O_4_, and C_14_H_25_O_4_ from this ion led to the formation of ions with *m/z* values of 263, 69, 61, and 60, respectively. The loss of C_9_H_18_O_6_ from the parent ion produced an ion with an *m/z* of 95, which was further fragmented to form an ion with an *m/z* of 81 by removal of methylene (CH_2_). In another pathway, the loss of C_9_H_18_O_6_ led to the formation of an ion with an *m/z* of 95. While an ion with an *m/z* of 67 was formed from the ion with an *m/z* of 95 by removal of C_2_H_4_. Removal of water from the parent ion produced an ion with an *m/z* of 299, this was followed by the loss of another water molecule to form an ion with an *m/z* of 281. The removal of CH_4_O_2_ from the parent ion formed an ion with an *m/z* of 263, which was further fragmented to produce an ion with an *m/z* of 251 by the removal of water.

Compound **3**, a phenolic compound with two hydroxyl groups in its structure, contains cyclic hydrocarbon with methylene and methyl. In the ESI positive ionization mode, compound **3** was protonated at the oxygen atom of one of the hydroxyl groups to form the parent ion [M + H]^+^ with an *m/z* of 349. The cyclic rearrangement and removal of C_4_H_6_, C_5_H_8_, C_6_H_10_, and C_10_H_18_ from the parent ion produced ions with an *m/z* of 295, 281, 267, and 211, respectively. Removal of a water molecule from the parent ion produced an ion with an *m/z* of 331, which was further fragmented to produce an ion with an *m/z* of 313 by removal of another water molecule. The loss of C_3_H_4_ from the parent ion led to the formation of an ion with an *m/z* of 309, this was followed by the loss of C_14_H_11_O to form an ion with an *m/z* of 114.

### 2.4. Molecular Docking

Molecular docking was performed on the three compounds that were identified using LC-MS based metabolomics to identify a potential binding mode of the compounds that could justify their inhibitory activity. Table 2 shows the binding affinities of the control ligand, the detected bioactive compounds and the standard competitive inhibitor (quercetin) towards the AG enzyme. A control docking procedure was performed using the co-crystallized control ligand, alpha-D-glucose (ADG) in order to validate the docking parameters, while quercetin was used as a comparison with the identified compounds. In this work, the complex with a more negative value (i.e., stronger binding affinity) is considered as the best-docked complex. The re-docked ADG was found to bind with 3A4A in a manner identitical to its crystallographic configuration. The value of the root mean square deviation (RMSD) of the re-docked ADG was found to be 0.633 Å, suggesting that the chosen docking parameters are able to reproduce the crystallised conformation. The docking parameters are considered to be acceptable if the RMSD value of the redocked ligand, with respect to the crystallised one, is less than 1.5 Å [36]. The control docking showed that the control ligand exerts a binding affinity of −6.0 kcal.mol towards the enzyme. Seven amino acid residues (ASP352, GLH277, ASH215, HIE112, ASH69, ARG442, HIE351) were involved in the hydrogen bond interactions with ADG. For quercetin, it exerts the binding affinity of −8.4 kcal/mol. The binding is assisted by two residues (ASH215, GLH277) via a hydrogen bond and three residues (PHE303, ASP352, and ARG442) via other types of interactions.

**Figure 5:**
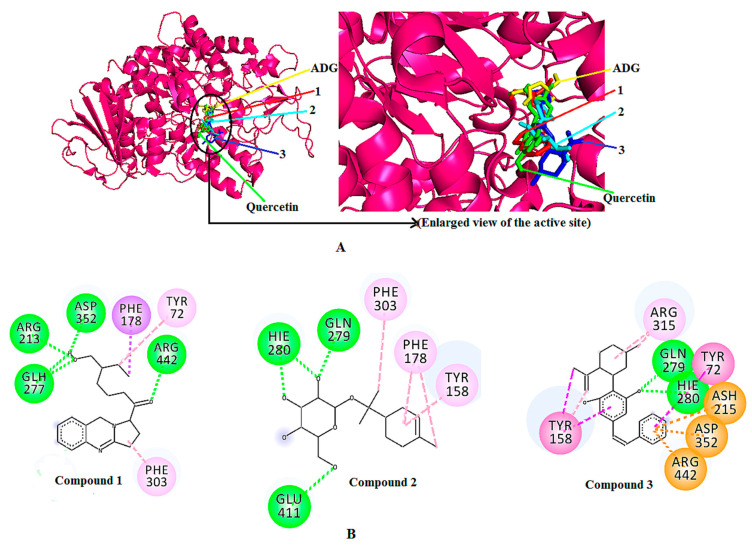
(**A**) Superimposed 3D diagram of control ligand (ADG), positive control (quercetin), the docked compounds **1**, **2**, and **3**. (**B**) 2D binding interactions of the docked compound **1**, **2,** and **3** with *α*-glucosidase (3A4A).

All of the three identified compounds exerted greater binding affinity towards the enzyme than that of the control ligand. Among the three identified compounds, compound **3** showed the highest binding affinity towards the enzyme, with -10.00 kcal/mol. It also had a higher binding affinity value when compared to quercetin (−8.4 kcal/mol). In contrast, compounds **1** and **2** showed binding affinities of −8.3 and −7.6 kcal/mol, respectively, which are lower than that of quercetin. The superimposed 3D docking visualization (Figure 5A) describes the predicted binding site of the three identified compounds, quercetin, and the control ligand (ADG) in the enzymatic protein. According to the figure, all compounds are predicted to bind at domain A of the enzyme, where the catalytic site is located. The same site is also occupied by ADG and quercetin, suggesting that a similar inhibition mechanism might be followed by the identified compounds.

Table 3 shows the binding interactions of compounds **1**, **2**, and **3**. Along with the H-bond interaction, other interactions such as pi-sigma, pi-alkyl, pi-pi t-shaped were also involved in the docked complexes. The binding interaction analysis showed that the hydroxyl moiety of compound **1** interacts with ARG213, ASP352 and protonated GLU277 residues, whereas the carbonyl moiety of compound **1** interacts with the ARG442 residue through hydrogen bond interaction. Among the four hydrogen bonds, the strongest hydrogen bond is the one involving ASP352 with a distance of 2.08 Å. Meanhile, TYR72 and PHE178 interact with the aliphatic chain of compound **1** through pi-alkyl and pi-sigma interactions, respectively. Whereas, the pyrrolidines moiety of compound **1** exhibited a pi-alkyl interaction with the PHE303 residue of *α*-glucosidase.

In the complex containing compound **2**, the tetrahydronpyran moeity of the compound was observed to form two hydrogen bonds with HIE280, with distances of 2.28 Å and 2.45 Å, respectively. The other two hydrogen bonds were established between the tetrahydropyran moiety of compound **2** and GLU411, and GLN279. Whereas, the alicyclic moiety of compound **2** interacted with TYR158 and PHE178 via pi-alkyl interactions. Furthermore, the aliphatic moiety of compound **2** interacted via a pi-alkyl bond with the PHE303 residue at a distance of 4.59 Å.

In the docked complex of compound **3**–AG enzyme, the hydroxyl moiety of the aromatic ring of compound **3** showed interactions with HIS280 and GLU279 residues through hydrogen bonding at distances of 2.78 Å and 2.58 Å, respectively. The aromatic ring of the phenolic and the aliphatic moieties of compound **3** were observed to involve pi–pi t-shaped and pi–alkyl interactions with TYR158. On the other hand, ASP352 and protonated ASP215 showed a pi–anion, and ARG442 showed a pi–cation interaction with the aromatic segment of compound **3**. Furthermore, the aromatic moiety of compound **3** also interacted with the TYR72 residue through a pi–pi t-shaped interaction. ARG315 interacted with the alicyclic moiety of compound **3** through two alkyl interactions at a distance of 4.50 Å and 3.84 Å (Table 3). The two-dimensional (2D) binding interactions of compounds **1**, **2**, and **3** with the active site of the 3A4A enzyme are depicted in Figure 5B.

## 3. Discussion

Various extraction methods using a methanolic solvent have been applied to enhance the content of the bioactive compounds possessing anti-diabetic and antioxidant activities in medicinal herbal extracts. Kidane et al. [37] reported that the extraction of *Psiadia punctulata* leaves in a methanolic solvent produced an extract with high AGI activity. A similar result was found by Bhatia et al. [38] who reported the high AGI activity of a methanolic extract of *Cornus capitata* Wall leaves. Extraction with aqueous methanol was shown to be better for the recovery of the largest amount of phenolic compounds from *Moringa oleifera* and rice bran, which are related to antioxidant activity [39,40]. In another study, 80% methanol solvent was used to extract antioxidants from numerous natural sources such as rice bran, wheat bran, oat groats and hull, coffee beans, citrus peel and guava leaves [41]. In this study, different ratios of aqueous methanolic solvent were investigated, which led to obtaining an extract with high AGI activity in 100% methanol. The trend of AGI activity (100 > 25 > 50 > 0 > 75% *v/v* methanol–water) is caused by the difference of metabolite profiling among the extracts which were affected by the solvent used during extraction [42,43,44,45].

The loading scatter plot obtained using OPLS (Figure 3B) pinpointed three putative compounds correlating to the AGI activity of *P. malayana* leaves. The presence of these compounds in this plant had not been recorded elsewhere; thus, this study reports it for the first time. The presence of compound **1** has been reported by Sutradhar et al. [28], who isolated it from the methanol and water extract of *Sida cordifolia*. Linn. aerial sections. This compound was reported to possess analgesic and anti-inflammatory effects in an animal model [27]. Although no research has been performed on the anti-diabetic effect of this compound, the plant (*S. cordifolia*) that is rich in this compound has been shown to ellicit both hypoglycemic and antioxidant activities [46,47].

Compound **2** is commonly known as *α*-terpineol-*β*-D-*O*-glucopyranoside or *α*-terpinyl-*β*-glucoside. Nhiem et al. [31] isolated this compound from a methanol extract of *Acanthopanax koreanum* leaves, although it did not possess antioxidant activity. This compound is also found in other plant parts such as apricot fruits [29], sweet potatoes [30], the aerial parts of *Ligularia alticola* [32], and an ethyl acetate extract of *Saccocalyx satureioides* [33].

Compound **3** was isolated from the stem bark of *Machaerium multiflorum* [34]. However, no anti-diabetic activity has been reported so far for this compound. Most of the reports indicate its antimicrobial activity against *Candida albicans*, *Cryptococcus neoformans*, *Mycobacterium intracellularae*, *Aspergillus fumigatus* [35], and *Staphylococcus aureus* [34].

This present study appears to be the first to investigate the potential anti-diabetic effect of these compounds through an in silico approach. The best method to confirm the AGI activity of these compounds is through an in vitro AGI test on each of the pure compounds. However, these compounds are not commercially available, and the isolation of these compounds is difficult due to their low abundnace in this plant. Thus, in silico computational analysis, evaluating the binding characteristic between the identified compounds and the active site of the enzyme, was carried out.

The *S. cerevisiae* isomaltase is made up of 589 amino acids. It consists of three domains, namely domain A (1–113 and 190–512), domain B (114–189) and domain C (513–589) [48]. There are three catalytic residues ASP215, GLU277 and ASP352, which are situated on the side of the C-terminal of domain A [17,48]. The catalytic residues indicate the active site of the enzymatic protein and all the residues involved in the molecular interaction during re-dock of ADG with 3A4A, contributing the actual catalytic reaction [17]. Based on the results obtained, all compounds demonstrated binding affinities comparable to that of the known AG inhibitor, quercetin, suggesting their potential to inhibit the enzyme.

Compounds **1** and **2** showed higher binding affinities than ADG, but slightly lower binding affinities compared to that of quercetin. Good binding affinity of these compounds to the active site of the enzyme was indicated, which is the same site used by ADG [17]. Among the seven residues that are involved in the binding of ADG towards the AG enzyme, three residues (ASP352, GLH277, and ARG442) were involved in the interactions with compound **1**, however, no active site residues of the control ligand interacted with compound **2**. This might explain the difference in the binding affinity (0.7 kca/mol) between compound **1** and compound **2**. It is anticipated that once the enzyme’s active site is blocked, the enzyme is unable to break down carbohydrates, resulting in lower quantities of glucose absorption and eventually lower blood glucose levels [10].

Compound **3** showed the highest binding affinity among the three compounds, quercetin, and ADG. This high binding affinity is attributed to its chemical structure which contains three main parts (aromatic, alicyclic, and aliphatic parts). This allows the compound to interact with three catalytic residues (ASP352, ARG442, and ASH215) via pi–anion and pi–cation interactions, in addition to hydrogen bond interactions. The higher number of hydrophobic interactions between compound **3** and the enzyme may lead to a high affinity towards the active site of the enzyme, which is in line with previous research [49]. Ahmed et al. [49] reported a higher number of hydrophobic interactions between questin (an identified *α*-glucosidase inhibitor) and 3A4A that indicated a high affinity towards the enzyme’s active site by exhibiting a more negative binding affinity value of −8.3 kcal/mol. Three residues (ASP352, ASH215, and ARG442) that are present in the ADG-AG enzyme complex are also involved in the docked complex of compound **3**–AG enzyme, supporting the high binding affinity exerted by compound **3**. Given the above findings, it is anticipated that compound **3** may serve as potential lead compound for the development of an AG inhibitor.

## 4. Materials and Methods

### 4.1. Materials

Organic chemicals (methanol, dimethyl sulfoxide) of analytical and chromatography grade and the AG (yeast maltase) enzyme were obtained from Merck (Darmstadt, Germany) and Megazyme (Megazyme, Bray, Ireland), respectively. Quercetin as the standard and *ρ*-nitrophenyl-*ρ*-D-glucopyranosidase (PNPG) as the substrate were procured from Sigma-Aldrich (St. Louis, MO, USA).

### 4.2. Plant Sample Collection

The *P. malayana* Jack leaves were collected from Cermin Nan Gedang at Sarolangun district, Jambi, Indonesia. These were identified by a taxonomist, Dr Shamsul Khamis from the University of Putra, Malaysia. The specimen was deposited at the Kulliyyah of Pharmacy (KOP) Herbarium, IIUM, Kuantan (voucher specimen #PIIUM008-2). The plant samples were allowed to dry for seven days at room temperature (25 ± 5 °C) in the shade and were converted into powder form using a Universal cutting mill (Fritsch, Idar-Oberstein, Germany). The powdered leaves were stored at −80 °C until further analysis [50].

### 4.3. Preparation of P. malayana Leaves Extracts

Crushed powdered leaves of *P. malayana* (1 g) were subjected to extraction. The extraction was conducted by sonication. The powdered leaves were immersed in 30 mL of methanol–water at various ratios (0, 25, 50, 75, and 100%, *v/v*) and allowed to sonicate for 30 min. Subsequently, the extracts were filtered using Whatman filter paper No.1. A rotary evaporator was used at 40 °C to remove the solvent. To clear any residual solvents, the extracts were then freeze-dried and stored at −80 °C. For this study, a total of 20 samples were prepared (4 replicates for each of the 5 separate extracts).

### 4.4. AGI Assay

The method reported by Javadi et al. [50] was followed with minor modifications. Quercetin (2 mg in 1 mL DMSO) and *ρ*-nitrophenyl-*ρ*-D-glucopyranosidase (PNPG, 6 mg in 20 mL of 50 mM phosphate buffer, pH 6.5) were used as the positive control and substrate, respectively. The yeast, glucosidase, was used in this study under the consideration that its substrate specificity, inhibitor sensitivity, and optimum pH are similar to the mammalian glucosidase [51].

The sample was prepared following the same preparation protocol for quercetin. While the negative control was prepared by replacing the sample with DMSO. A total of 10 µL of the samples, quercetin and DMSO; 100 µL of 30 mM phosphate buffer and 15 µL of 0.02 U/µL AG enzyme were transferred into 96-well plate. A blank was prepared following the same protocol but without the enzyme. The samples and the blank mixture were both incubated at room temperature for 5 min and then treated with 75 μL of PNPG, followed by addition of glycine (pH 10) and another incubation time of 15 min at room temperature to stop the reaction. The absorbance reading was recorded at 405 nm by the microplate reader (Tecan Nanoquant Infinite M200, Männedorf, Switzerland). The inhibition percentage was obtained from the following equation:

Inhibition activity (%) = [(A_control_ − A_sample_)/A_control_] × 100% (1)

where, the absorbance of the negative control is A_control_, and the absorbance of a sample or positive control is A_sample_.

### 4.5. LCMS-QTOF Analysis

The method used by Murugesu et al. [17] was followed in this study with a slight modification. LCMS-QTOF instrument (Agilent 1290 Infinity and 6550 iFunnel, Santa Clara, CA, USA) fitted with an electrospray interface (ESI) with positive ion modes was used to analyze the sample. The sample was prepared using 250 μL of methanol to dissolve 1 mg of each plant extract. The mixture was vortexed and sonicated for 15 min, followed by the addition of 250 µL of water. Subsequently, it was centrifuged for 15 min to obtain a clear supernatant which was transferred into an insert glass vial through syringe filtration. Phenomenex Kinetex C18 core-shell technology 100 Å (250 mm × 4.6 mm, 5 μm, California, United States) column was used for sample injection. The column temperature was 27 °C. The column was initially washed with methanol before sample injection. A total of 10 µL sample was injected through an autosampling system. Samples were eluted with a gradient system from 5% methanol in water with 0.1% formic acid to absolute methanol in 20 min. The machine was then operated for another 10 min with absolute methanol at a flow rate of 0.7 mL/min for a total running time of 30 min. The MS/MS data were recorded between *m/z* values of 50 to 1500 with a scanning rate of 1 spectrum per scan, and the MS/MS spectra were collected by a collision energy ramp of 35 eV. The source parameter was set to the following: gas temperature = 200 °C, gas flow = 14 L/min, nebulizer= 35 psig, sheat gas temperature = 350 °C, sheat gas flow = 11 L/min.

ACD/Spec Manager v.12.00 (Advanced Chemistry Development, Inc., ACD/Labs Toronto, ON, Canada) software was used for analyzing the acquired LCMS-QTOF data and for in silico analysis of fragmention pathway. The raw (*.xms) data were converted into CDF (*.cdf) format by ACD/Spec manager. The data were subsequently pre-processed using MZmine software (VTT Technical Research Centre, Oulu, Finland) for baseline correction, peak detection, peak filtering, alignment, smoothing and gap filling and saved as a (*.csv) format [52] following the parameters shown in Table 4. The results were analyzed with SIMCA-P^+^ 14.0 (Umetrics, Umeå, Sweden) software for multivariate data analysis.

### 4.6. Molecular Docking

Docking was carried out using AutoDock Vina (version 1.1.2) to predict the binding affinity of the identified compounds to the active site of the enzyme. From Protein Data Bank (PDB), the AG crystal structure was combined (PDB code: 3A4A) [17,53,54,55,56,57]. The 3D structures of the identified compound were collected from the Super Natural II database as *.mol files and quercetin was collected from Pub Chem in *.sdf format. All the structures of the identified compounds were changed to *.pdb format by Open Babel (version 2.3.1) [17]. Then AutoDock Tools (version 1.5.6) was used to convert the compounds in *.pdb format to *. Pdbqt, prior to docking. Gasteiger charges and hydrogen atoms were added [53]. The rotatable bonds were set using AutoDock Tools. The co-crystallized ligand, alpha-D-glucose (ADG), was re-docked into the enzyme (PDB ID: 3A4A). The top-levelled docking conformations were compared with the actual crystallographic conformation based on the values of root mean square deviation (RMSD). The same parameters as in the control docking were applied for the docking of quercetin, as well as compounds **1**, **2** and **3** against the enzyme [36,58]. The grid box parameters were centered at the coordinate of 21.272, −0.751 and 18.634. The sizes of the grid box for X, Y, and Z were 20 Å, 26 Å and 22 Å, respectively, while the exhaustiveness was set as 16. The docking was performed in triplicate. The enzyme crystal structure was protonated at a pH of 6.5 with the PDB2PQR Server, version 2.0.0, to simulate the real state of the bioassay [17,59]. The 3D superimposed diagram of the standard and the detected compounds were rendered using PyMOL TM 1.7.4.5 (Schrödinger, LLC, New York, NY, USA) [17]. All docking outcomes were analyzed by Biovia Discovery Studio Visualizer (San Diego, CA, USA) [17,36,58].

### 4.7. Statistical Analysis

Minitab 16 (Minitab Inc., State College, PA, USA) was used for the statistical analysis of the obtained data. One-way variances (ANOVA) and Tukey’s test were used to analyze the data with a significance level of *p* < 0.05. The data are interpreted as a mean ± standard deviation (SD). Multivariate data analysis was carried out using SIMCA P + 14.0 (Umetrics, Umeå, Sweden) software where the pareto scaling method was applied.

## 5. Conclusions

LC-MS-based multivariate data analysis and molecular docking is a promising technique for investigating bioactive compounds associated with folk medicine, for example those from herbal plants. In the present study, three AG inhibitor compounds were identified from methanolic extracts of *P. malayana* leaves by using this approach. The compounds were identified as 5′-hydroxymethyl-1′-(1, 2, 3, 9-tetrahydro-pyrrolo (2, 1-*b*) quinazolin-1-yl)-heptan-1′-one, α-terpinyl-*β*-glucoside, and machaeridiol-A, being reported for the first time in this plant in this study. Their AG inhibitory activity was confirmed through in silico molecular studies, which predicted the binding mode and binding interactions between the identified compounds and the binding site on the enzyme. This information is useful for future studies on the bioassay assessment of these compounds for exploration of their potential uses as novel anti-diabetic agents. Purification and identification of the unknown compounds suspected to possess AG inhibitory activities in this study are required to be conducted in the future study.

## Figures and Tables

**Table 1:** Tentative metabolites identified in the *P. malayana* Jack leaves through LC-MS/MS fragmentation using positive ionization.

Compound	M + H	MS^2^ Fragments Ion	Tentative Metabolites	Reference
**1**	315.1775 (C_19_H_27_N_2_O_2_)	[M-CH_2_]^+^ at *m/z* 300, [M-CH_3_]^+^ at *m/z* 299, [M-CH_5_]^+^ at *m/z* 297, [M-C_2_H_4_]^+^ at *m/z* 286, [M-CH_3_O]^+^ at *m/z* 283, [M-CH_4_O]^+^ at *m/z* 282, [M-C_4_H_2_]^+^ at *m/z* 264, [M-C_4_H_9_]^+^ at *m/z* 257, [M-C_4_H_9_O]^+^ at *m/z* 241, [M-C_6_H_3_]^+^ at *m/z* 239, [M-C_5_H_11_O]^+^ at *m/z* 227, [M-C_7_H_15_O]^+^ at *m/z* 199, [M-C_7_H_13_O_2_]^+^ at *m/z* 185, [M-C_9_H_19_O]^+^ at *m/z* 171, [M-C_10_H_21_O]^+^ at *m/z* 157, [M-C_12_H_15_O]^+^ at *m/z* 139, [M-C_11_H_18_O_2_]^+^ at *m/z* 132, [M-C_12_H_17_O_2_]^+^ at *m/z* 121, [M-C_14_H_21_O_2_]^+^ at *m/z* 93, [M-C_13_H_19_N_2_O_2_]^+^ at *m/z* 79, [M-C_17_H_23_N_2_O_2_]^+^ at *m/z* 27	5′-hydroxymethyl-1′-(1, 2, 3, 9-tetrahydro-pyrrolo (2, 1-*b*) quinazolin-1-yl)-heptan-1′-one	[27,28]
**2**	317.2882 (C_16_H_29_O_6_)	[M-HO]^+^ at *m/z* 299, [M-H_3_O_2_]^+^ at *m/z* 281, [M-CH_3_O_2_]^+^ at *m/z* 269, [M-CH_5_O_3_]^+^ at *m/z* 251, [M-C_4_H_5_]^+^ at *m/z* 263, [M-C_9_H_17_O_6_]^+^ at *m/z* 95, [M-C_10_H_19_O_6_]^+^ at *m/z* 81, [M-C_11_H_19_O_6_]^+^ at *m/z* 69, [M-C_11_H_21_O_6_]^+^ at *m/z* 67, [M-C_14_H_23_O_4_]^+^ at *m/z* 61, [M-C_14_H_24_O_4_]^+^ at *m/z* 60	*α*-terpinyl-*β*-glucoside	[29,30,31,32,33]
**3**	349.2127 (C_24_H_29_O_2_)	[M-HO]^+^ at *m/z* 331, [M-H_3_O_2_]^+^ at *m/z* 313, [M-C_3_H_3_]^+^ at *m/z* 309, [M-C_4_H_5_]^+^ at *m/z* 295, [M-C_5_H_7_]^+^ at *m/z* 281, [M-C_6_H_9_]^+^ at *m/z* 267, [M-C_10_H_17_]^+^ at *m/z* 211, [M-C_17_H_14_O]^+^ at *m/z* 114	machaeridiol-A	[34,35]

**Table 2:** Binding affinities of the *α-*glucosidase enzyme (3A4A) with control ligand, the known competitive inhibitor (quercetin), and the identified active compounds.

Compound	Binding Affinity, kcal/mol
Control ligand (ADG)	−6.0
Quercetin	−8.4
**1**	−8.3
**2**	−7.6
**3**	−10.0

**Table 3:** Data for the molecular docking of compounds **1**, **2**, and **3** in 3A4A.

Compound Structure	Interacting Amino Acid Residues	Bond Type	Bond Distance(Å)
Compound **1**	ASP352	Hydrogen bonding	2.08
	ARG213	Hydrogen bonding	2.82
	GLH277	Hydrogen bonding	2.60
	ARG442	Hydrogen bonding	2.64
	TYR72	Pi-Alkyl	4.49
	PHE178	Pi-Sigma	3.68
	PHE303	Pi-Alkyl	4.76
Compound **2**	GLN279	Hydrogen bonding	2.36
	HIE280	Hydrogen bonding	2.28 2.45
	GLU411	Hydrogen bonding	3.39
	PHE303	Pi-Alkyl	4.59
	PHE178	Pi-Alkyl	4.82 5.25
	TYR158	Pi-Alkyl	5.24
Compound **3**	GLN279	Hydrogen bonding	2.58
	HIE280	Hydrogen bonding	2.78
	ASP352	Pi-Anion	4.50
	ASH215	Pi-Anion	4.80
	ARG442	Pi-Cation	3.66
	TYR72	Pi-Pi T-shaped	5.30
	TYR158	Pi-Pi T-shaped Pi-Alkyl Pi-Alkyl	4.85 4.16 4.09
	ARG315	Alkyl	4.50 3.84

**Table 4:** Parameter setting for pre-processing of LC-MS data using MZmine software (VTT Technical Research Centre, Finland).

Parameters	Details
Baseline correction	Mass Spectrometry (MS) level = 1 *m/z* bin width = 1 Asymmetric baseline corrector (smoothing = 100,000, asymmetry = 0.5)
Mass detection	Mass detector = centroid (noise level = 200)
Chromatogram builder	Min time span = 0.2 min Min height = 200 *m/z* tolerance = 1.0 mz or 2500 ppm
Peak detection	Filter width = 11
Isotopic peaks grouper	*m/z* tolerance = 1.0 mz or 2500 ppm Retention time (RT) tolerance = 0.2 min Monotonic shape Maximum charge = 1
Duplicate peak filter	*m/z* tolerance = 1.0 mz or 2500 ppm RT tolerance = 0.2 min
Normalization	Linear Normalization Peak measurement type = peak area
Alignment (Join aligner)	*m/z* tolerance = 1.0 or 2500 ppm Weight for *m/z* = 85 RT tolerance = 0.2 min Weight for RT = 15 Require same charge state
Gap filling	Same RT and *m/z* range gap filler *m/z* tolerance = 1.0 or 2500 ppm

## Supplementary Materials

The following is available online, Figure S1: Fragmentation pathway of three identified compounds.
