# Supplementary material for: Characterization of α-Glucosidase Inhibitors from Psychotria malayana Jack Leaves Extract Using LC-MS-Based Multivariate Data Analysis and In-Silico Molecular Docking
Source: Molecules. 2020 Dec 12;25(24):5885. doi: 10.3390/molecules25245885 (PMC7763559; doi:10.3390/molecules25245885)
Supplement: Supplementary file 1 [file molecules-25-05885-s001.pdf]

**Figure S1.** Fragmentation pathway of three identified compounds.

1. **Compound 1 (315.1775):**

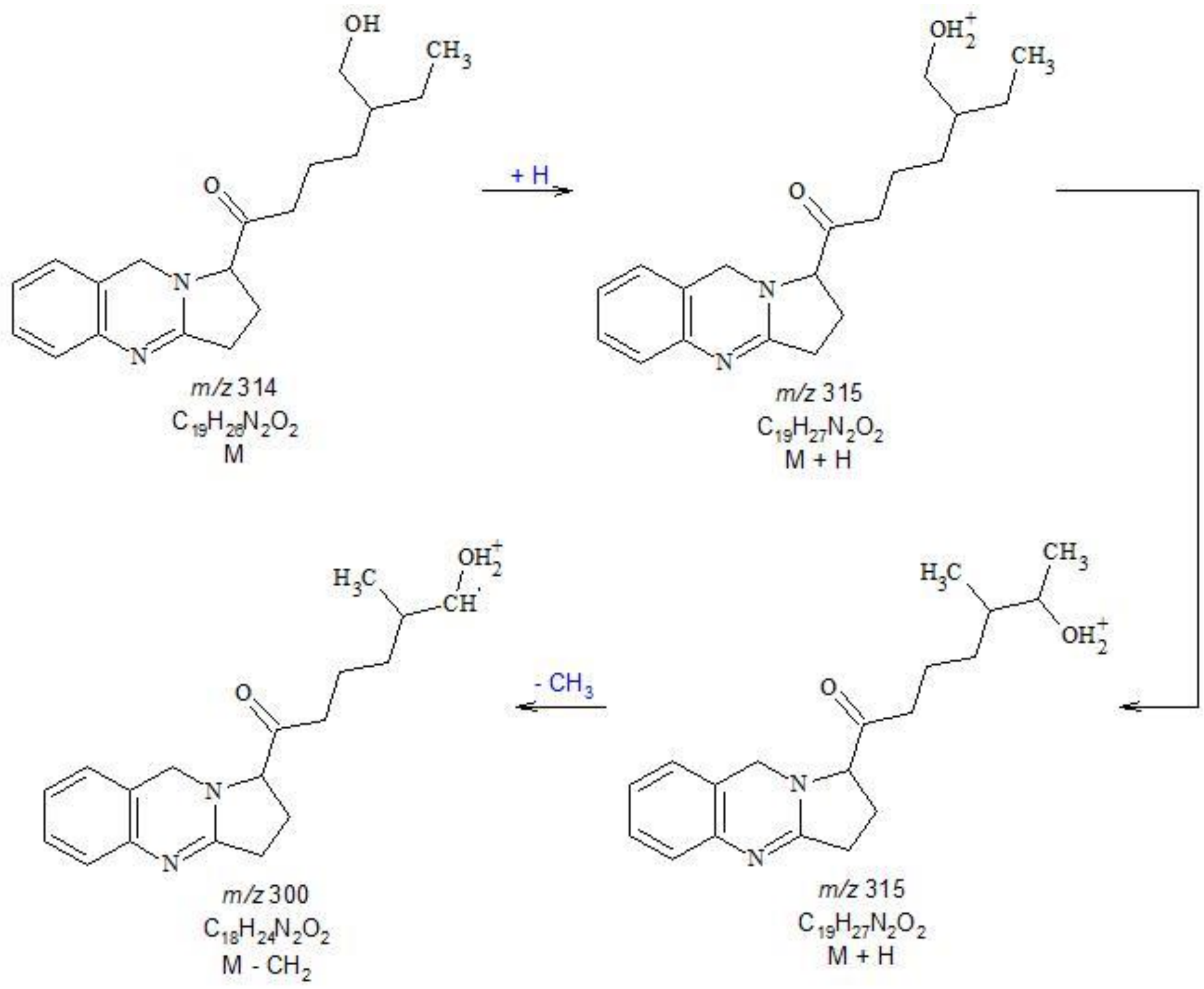

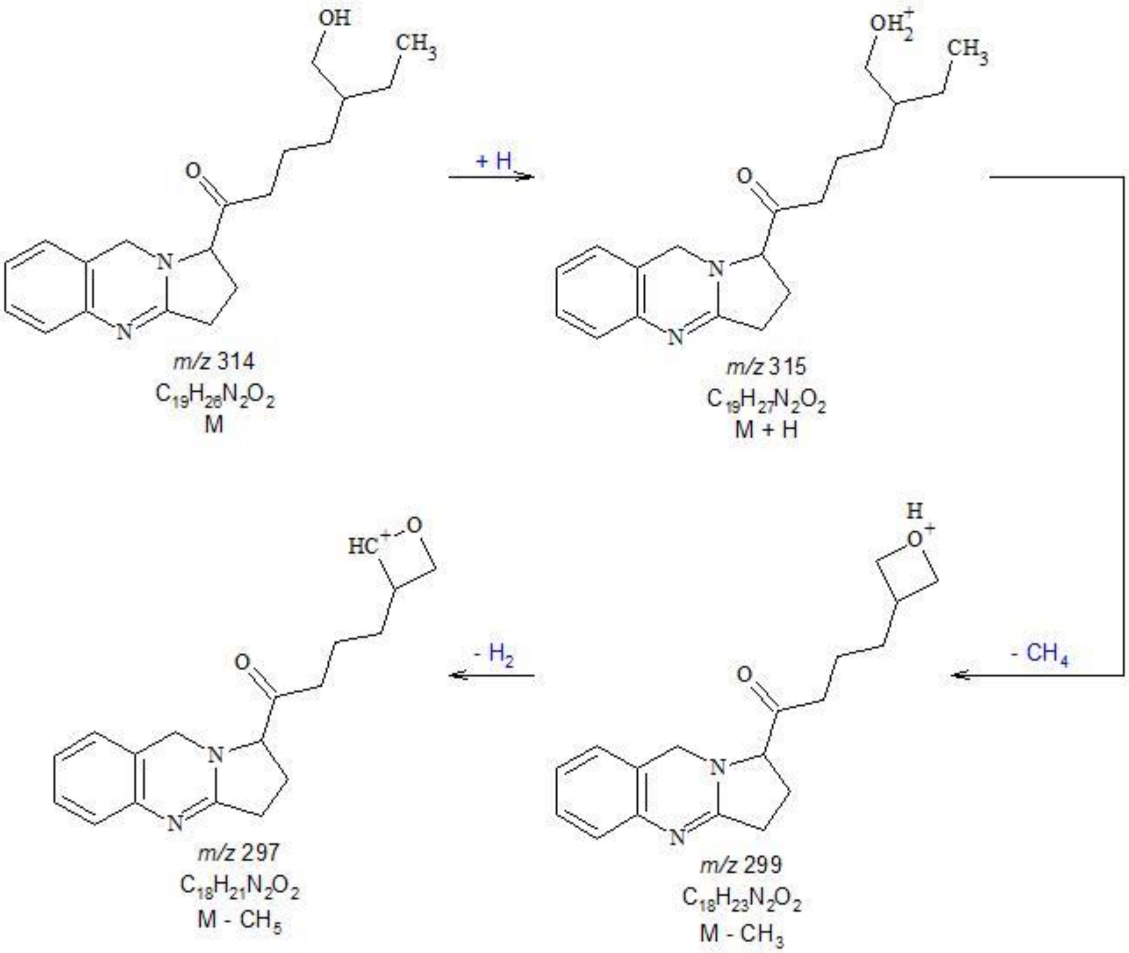
OH
CH3
O
N
N
m/z 314
C19H26N2O2
M
+ H
OH2+
CH3
m/z 315
C19H27N2O2
M + H
- CH4
H
O+
m/z 299
C18H23N2O2
M - CH3
- H2
HC+ O
m/z 297
C18H21N2O2
M - CH5

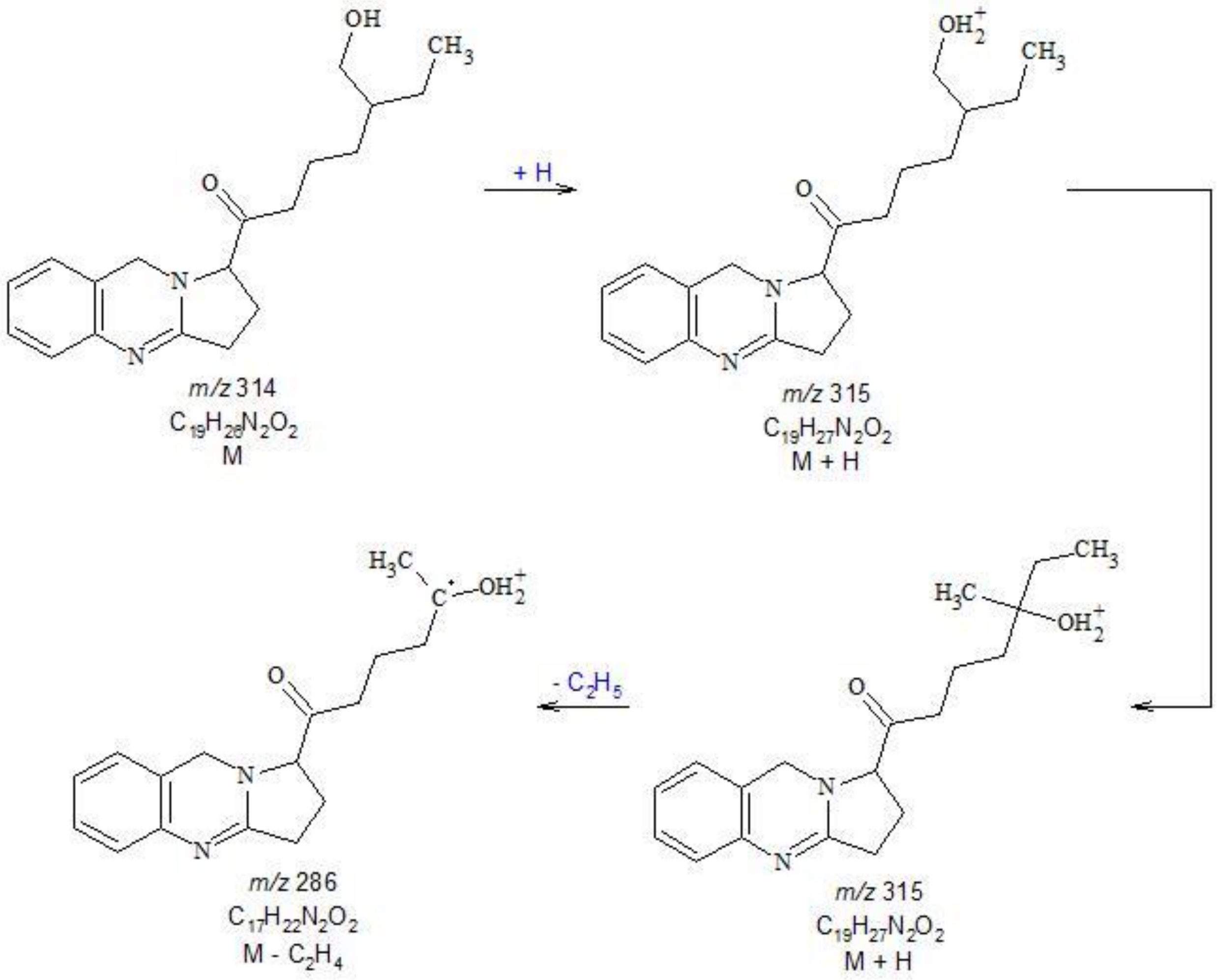
+ H
m/z 314
C19H26N2O2
M
m/z 315
C19H27N2O2
M + H
- C2H5
m/z 286
C17H22N2O2
M - C2H4
m/z 315
C19H27N2O2
M + H

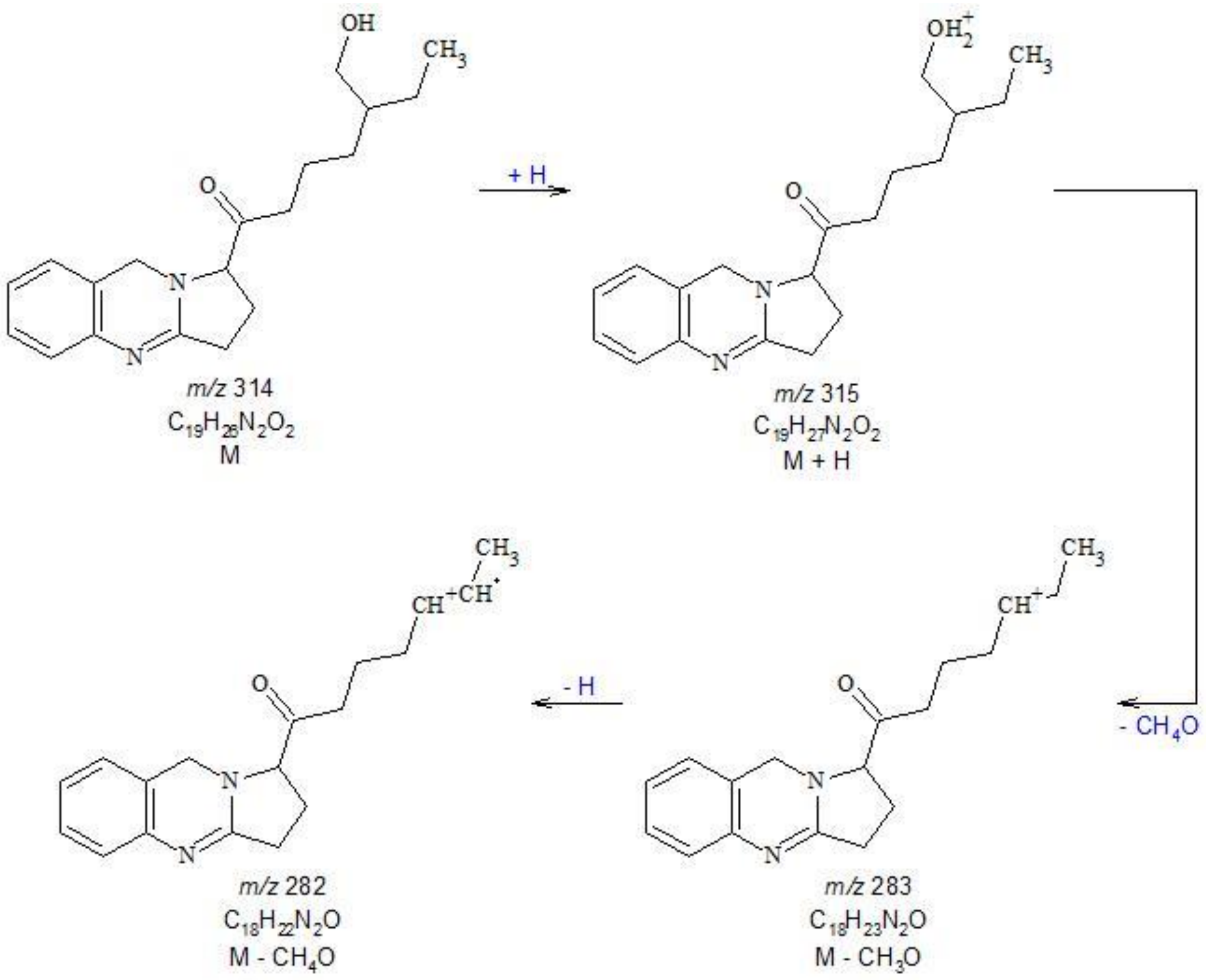
+ H
$m/z$ 314
$C_{19}H_{26}N_2O_2$
M
$m/z$ 315
$C_{19}H_{27}N_2O_2$
M + H
- $CH_4O$
$m/z$ 283
$C_{18}H_{23}N_2O$
M - $CH_3O$
- H
$m/z$ 282
$C_{18}H_{22}N_2O$
M - $CH_4O$

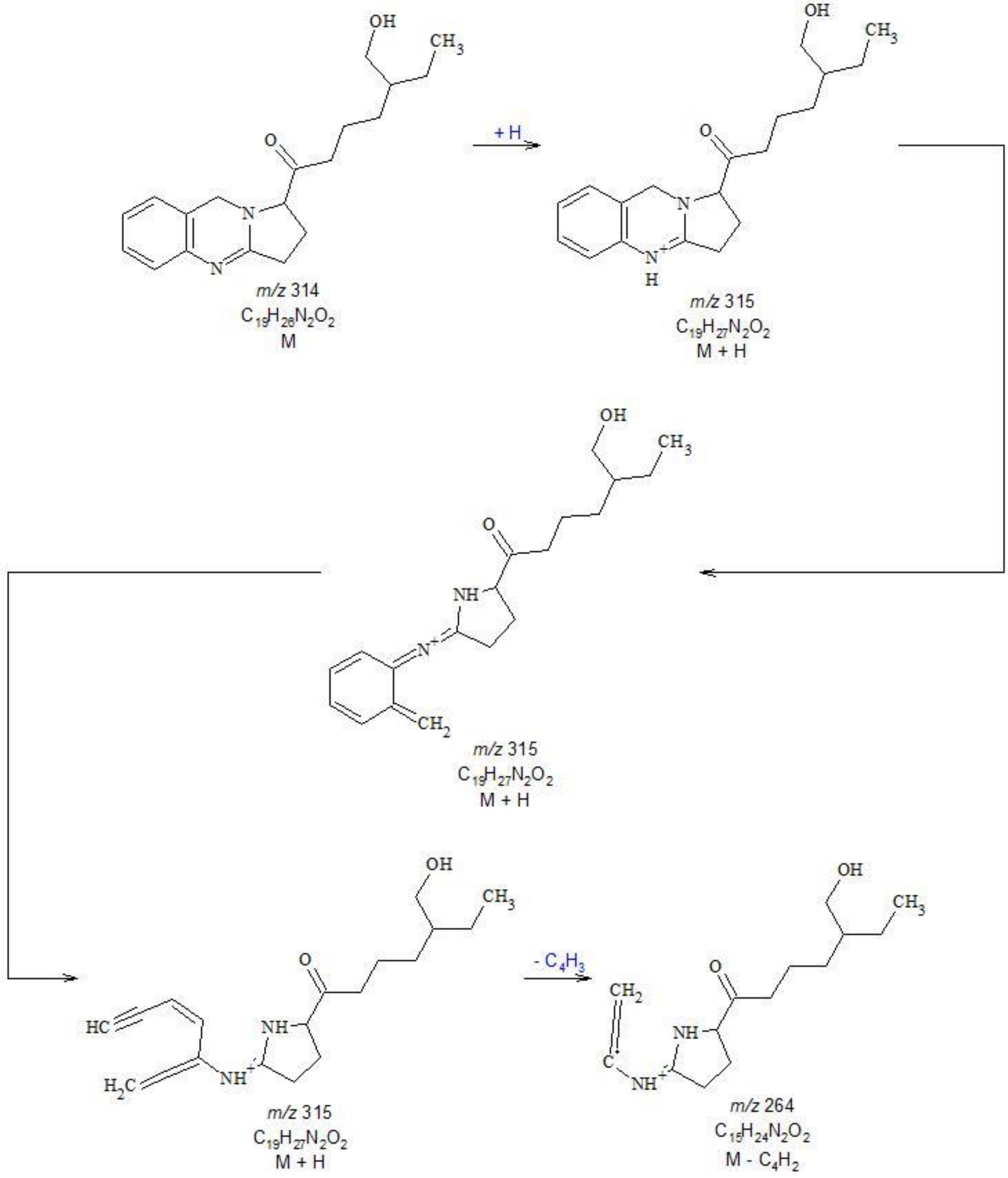

OH
CH3
O
N
N
m/z 314
C19H26N2O2
M
+ H
m/z 315
C19H27N2O2
M + H
NH
N+
CH2
m/z 315
C19H27N2O2
M + H
HC
H2C
NH+
m/z 315
C19H27N2O2
M + H
- C4H3
m/z 264
C15H24N2O2
M - C4H2

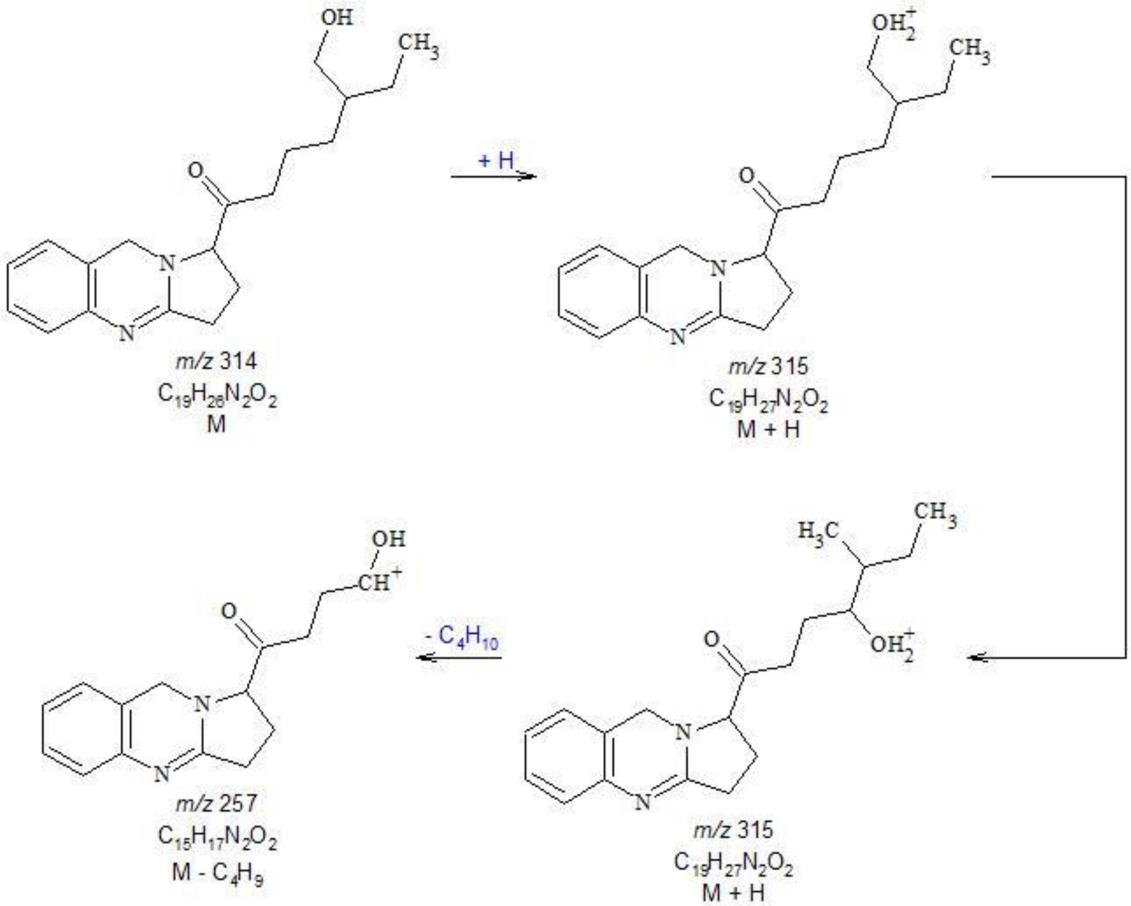
OH
CH3
+ H
OH2+
CH3
O
N
N
m/z 314
C19H26N2O2
M
m/z 315
C19H27N2O2
M + H
H3C
CH3
OH
CH+
- C4H10
OH2+
m/z 257
C15H17N2O2
M - C4H9
m/z 315
C19H27N2O2
M + H

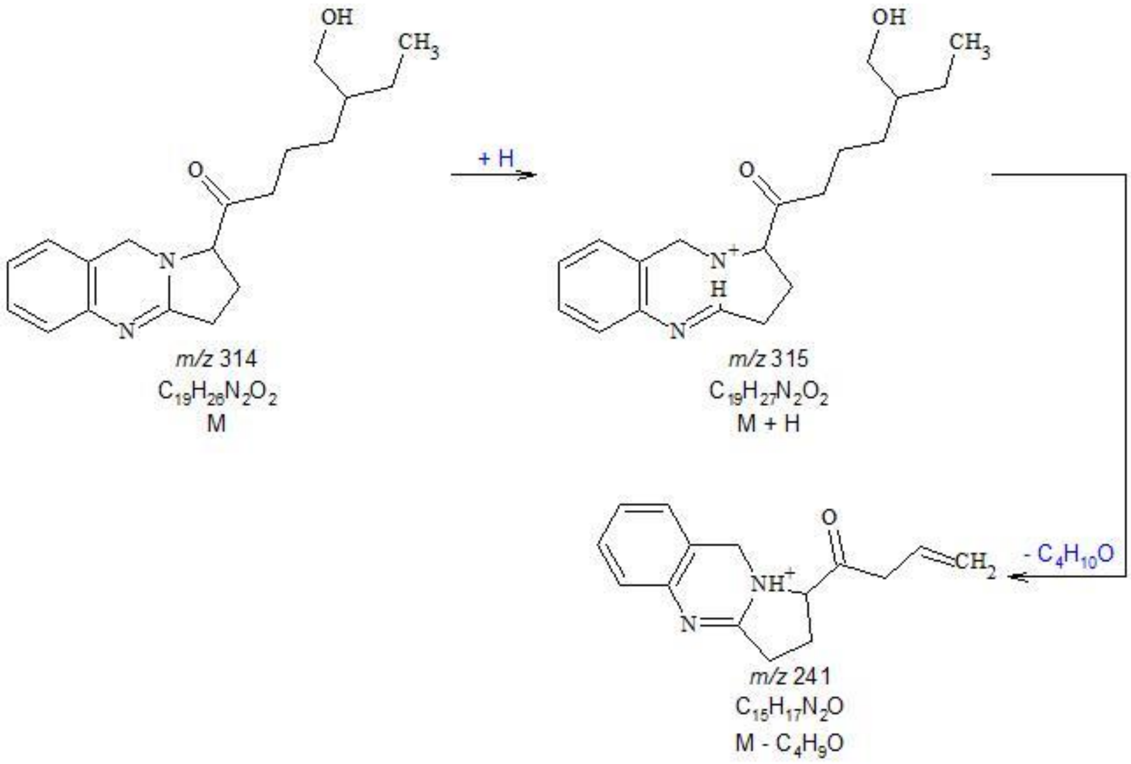
OH
CH3
O
N
N
m/z 314
C19H26N2O2
M
+ H
N+
H
m/z 315
C19H27N2O2
M + H
- C4H10O
NH+
CH2
m/z 241
C15H17N2O
M - C4H9O

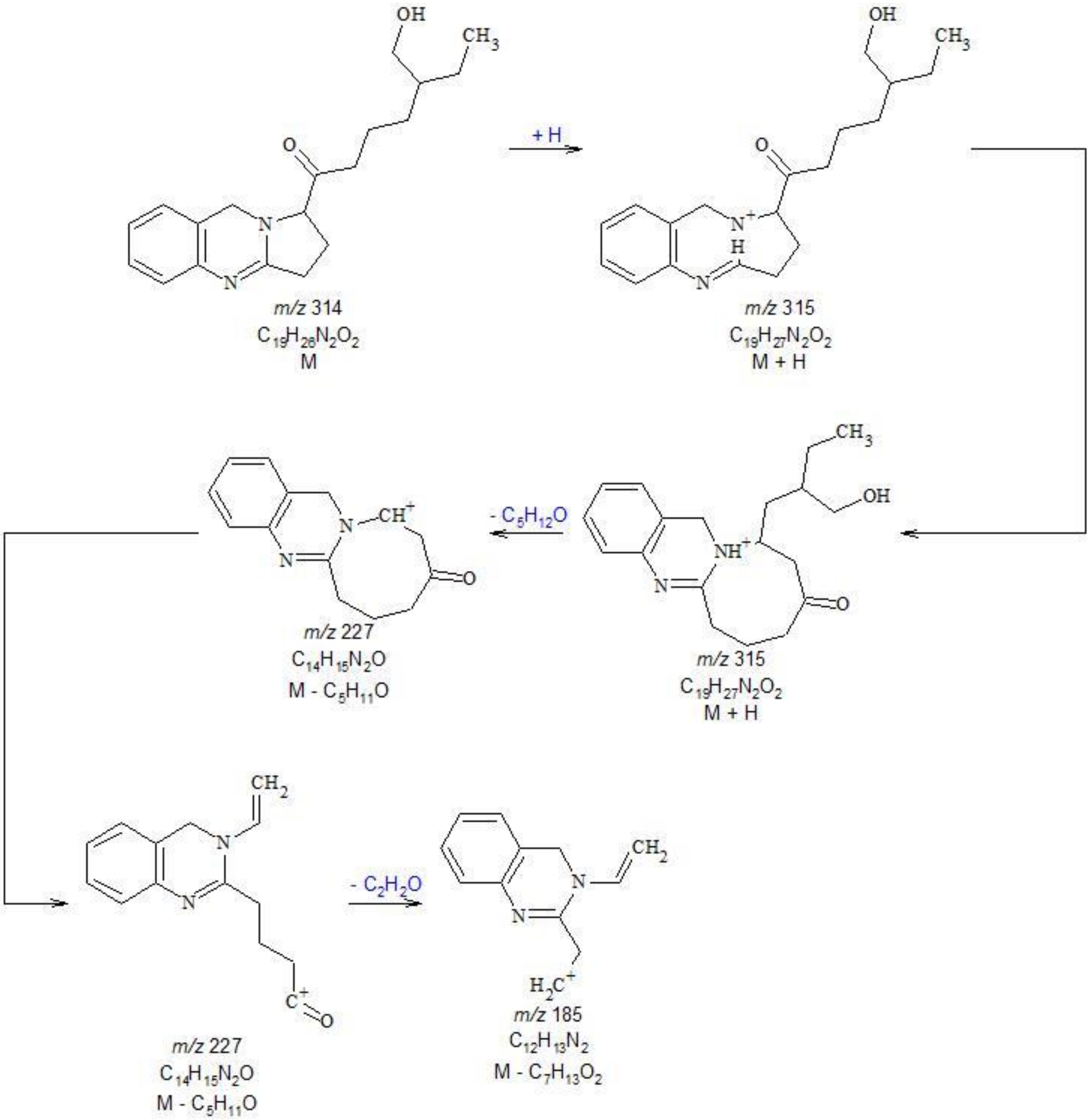
OH
CH3
O
N
N
m/z 314
C19H26N2O2
M
+ H
OH
CH3
O
N+
H
N
m/z 315
C19H27N2O2
M + H
CH3
OH
NH+
N
O
m/z 315
C19H27N2O2
M + H
- C5H12O
N
CH+
N
O
m/z 227
C14H15N2O
M - C5H11O
CH2
N
N
C+
O
m/z 227
C14H15N2O
M - C5H11O
- C2H2O
CH2
N
N
H2C+
m/z 185
C12H13N2
M - C7H13O2

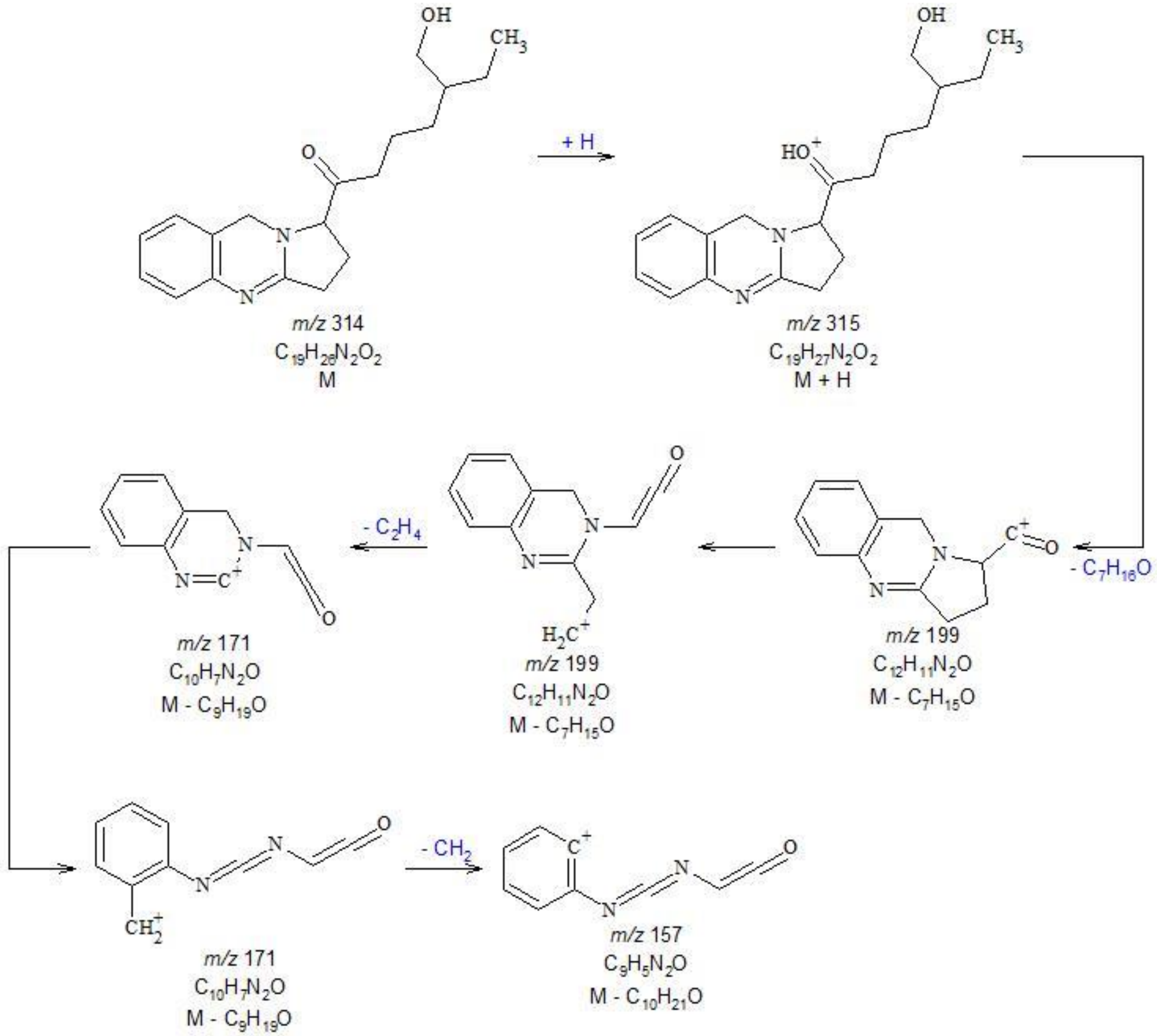

OH
CH3
O
N
N
m/z 314
C19H26N2O2
M
+ H
HO+
m/z 315
C19H27N2O2
M + H
- C7H16O
C+
O
m/z 199
C12H11N2O
M - C7H15O
H2C+
m/z 199
C12H11N2O
M - C7H15O
- C2H4
N=C+
m/z 171
C10H7N2O
M - C9H19O
CH2+
m/z 171
C10H7N2O
M - C9H19O
- CH2
C+
m/z 157
C9H5N2O
M - C10H21O

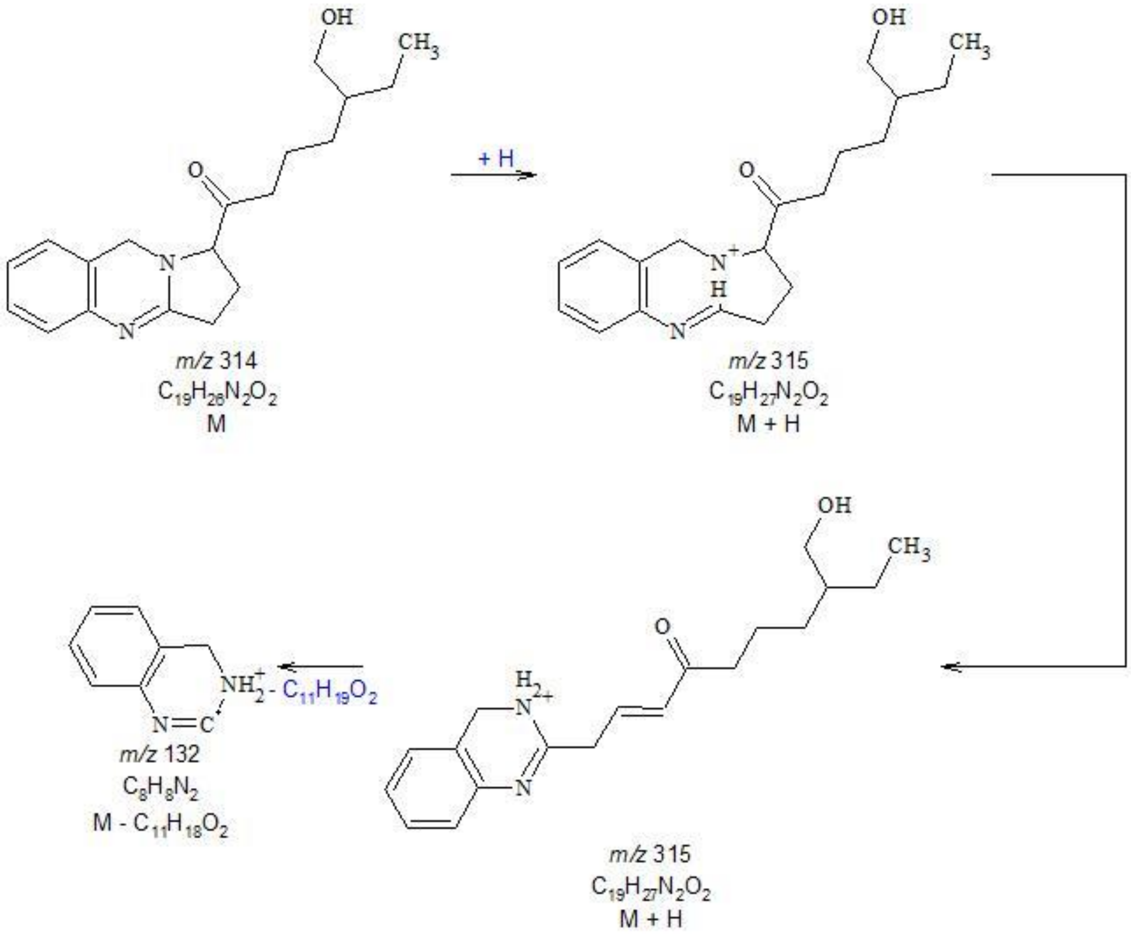
OH
CH3
O
N
N
m/z 314
C19H26N2O2
M
+ H
OH
CH3
O
N+
H
N
m/z 315
C19H27N2O2
M + H
OH
CH3
O
H2
N+
N
m/z 315
C19H27N2O2
M + H
NH2+
- C11H19O2
N=C
m/z 132
C8H8N2
M - C11H18O2

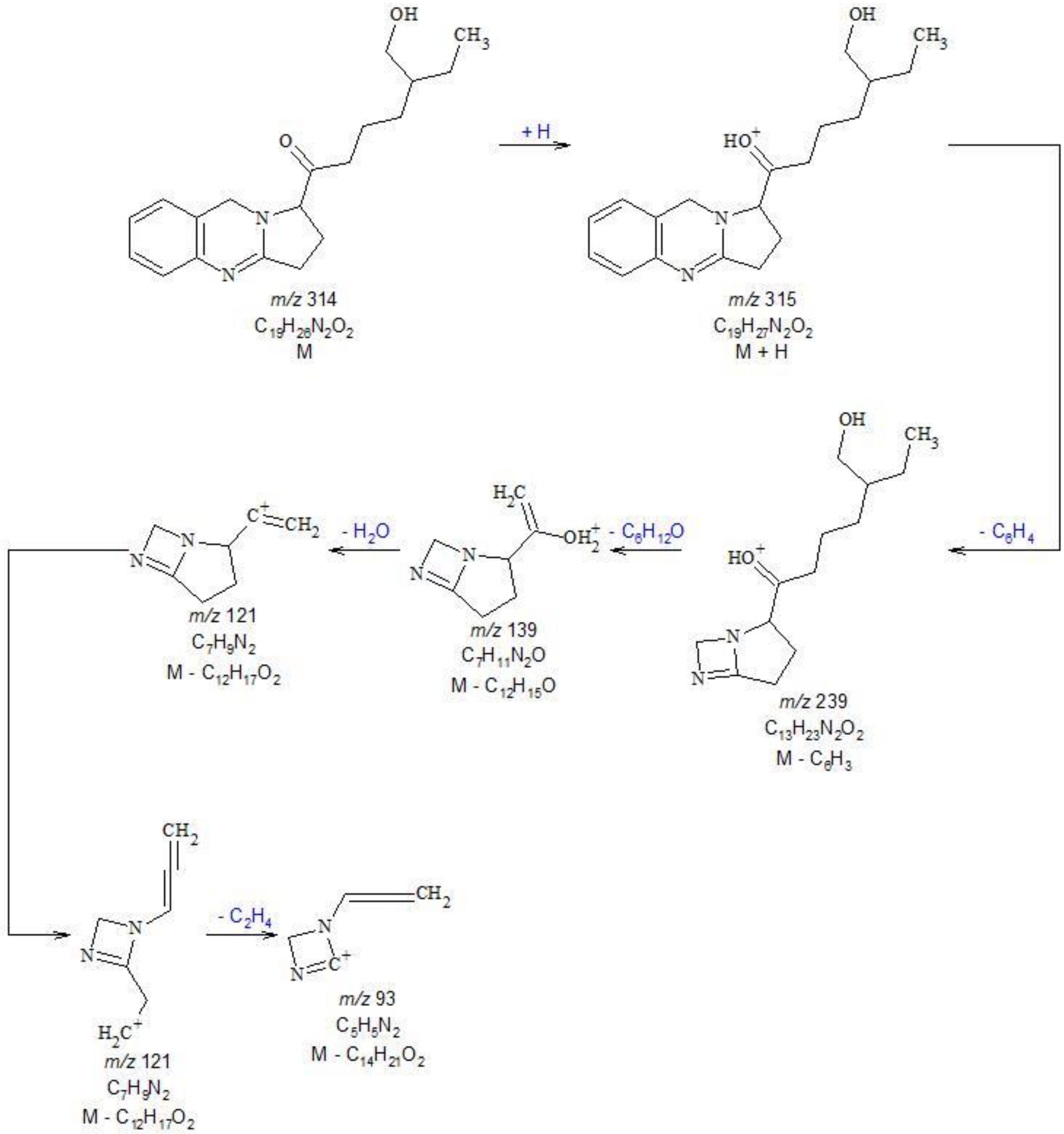
OH
$CH_3$
O
N
N
*m/z* 314
$C_{19}H_{26}N_2O_2$
M
+ H
$HO^+$
*m/z* 315
$C_{19}H_{27}N_2O_2$
M + H
- $C_6H_4$
*m/z* 239
$C_{13}H_{23}N_2O_2$
M - $C_6H_3$
- $C_6H_{12}O$
$H_2C$
$OH_2^+$
*m/z* 139
$C_7H_{11}N_2O$
M - $C_{12}H_{15}O$
- $H_2O$
$C^+$
$CH_2$
*m/z* 121
$C_7H_9N_2$
M - $C_{12}H_{17}O_2$
$CH_2$
$H_2C^+$
*m/z* 121
$C_7H_9N_2$
M - $C_{12}H_{17}O_2$
- $C_2H_4$
$C^+$
*m/z* 93
$C_5H_5N_2$
M - $C_{14}H_{21}O_2$

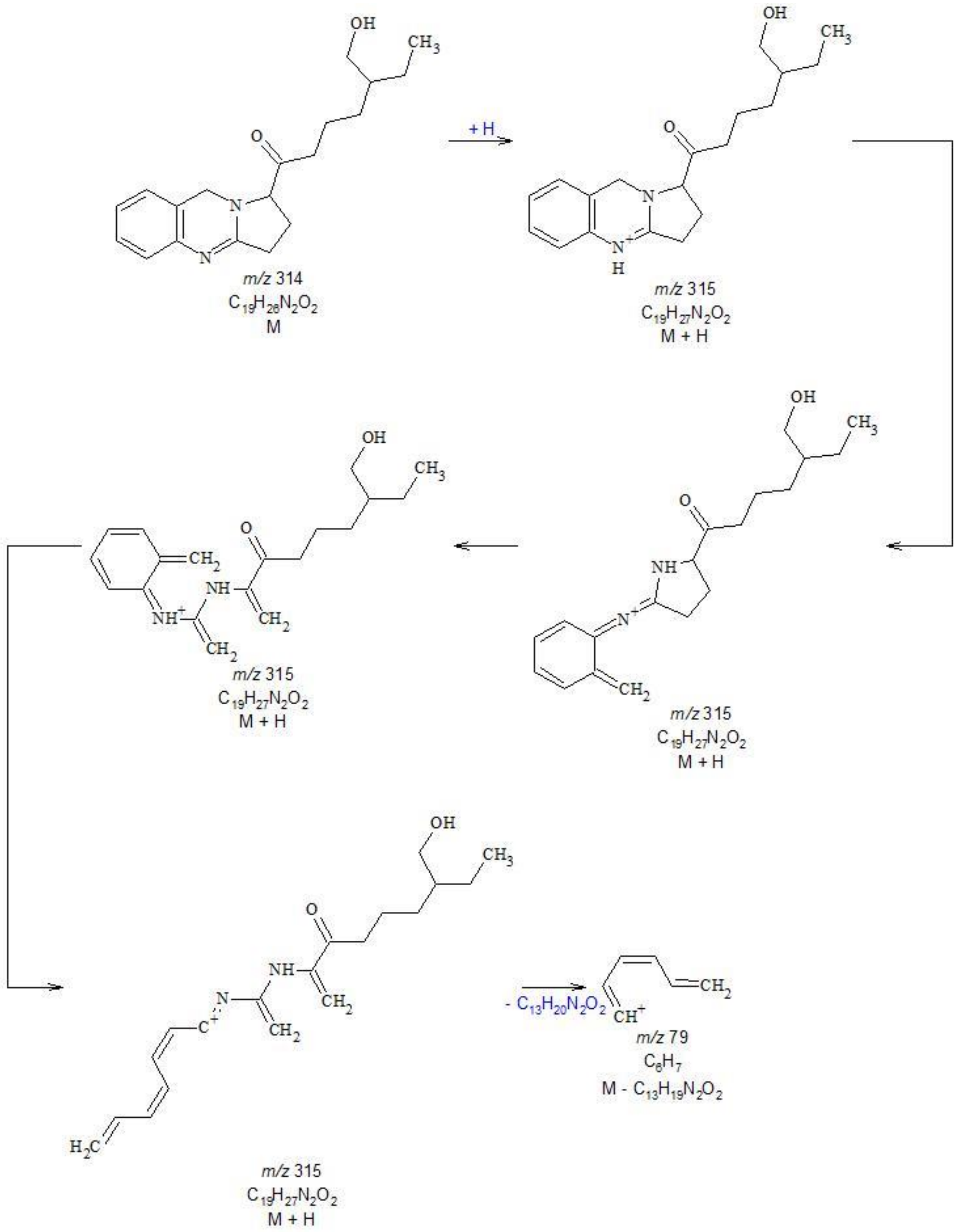
OH
CH3
O
N
m/z 314
C19H26N2O2
M
+ H
N+
H
m/z 315
C19H27N2O2
M + H
NH
CH2
NH+
m/z 315
C19H27N2O2
M + H
C+
- C13H20N2O2
CH+
m/z 79
C6H7
M - C13H19N2O2
H2C
m/z 315
C19H27N2O2
M + H

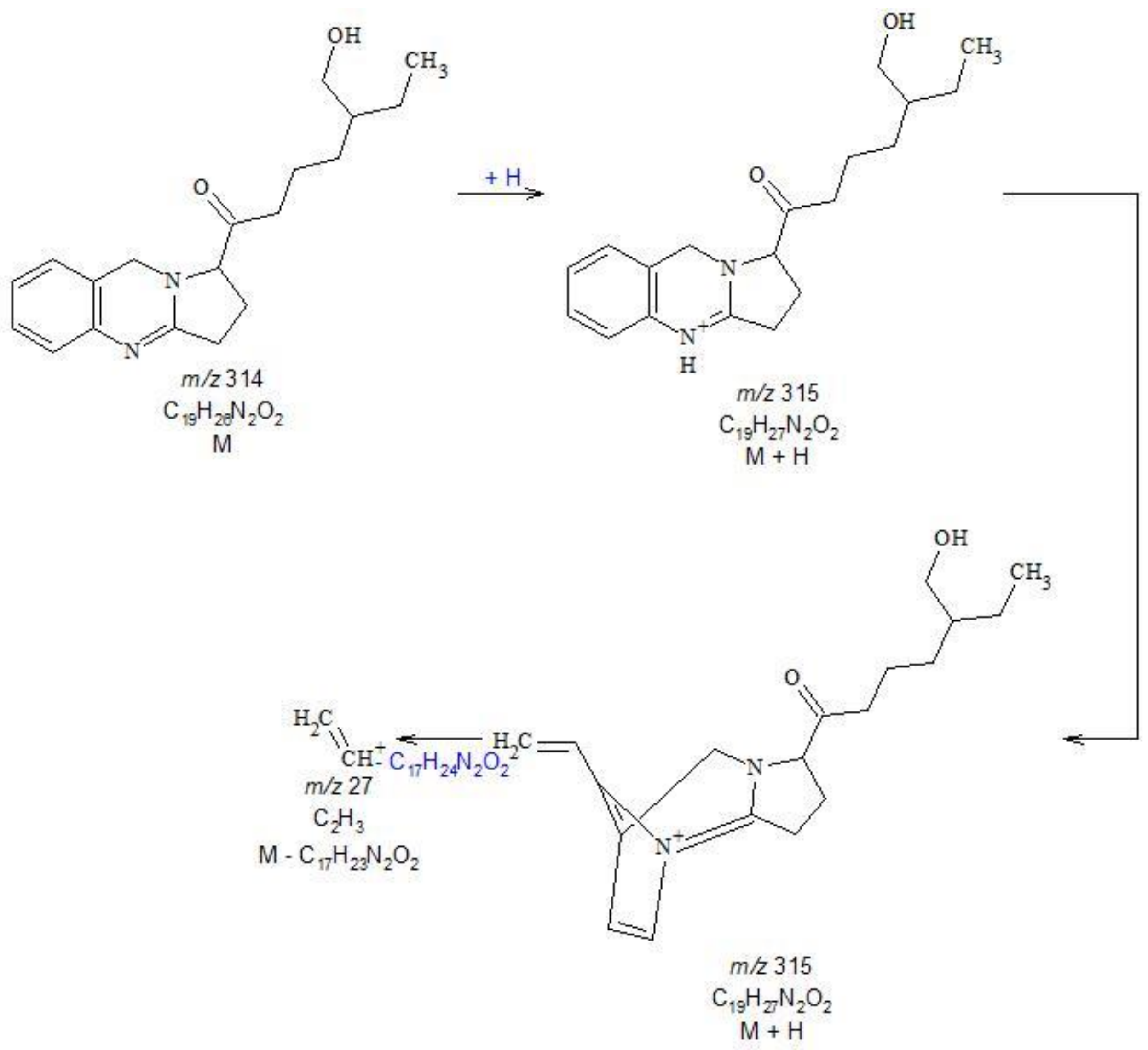
OH
CH3
O
N
+ H
N+
H
m/z 314
C19H26N2O2
M
m/z 315
C19H27N2O2
M + H
H2C
CH+
C17H24N2O2
H2C
m/z 27
C2H3
M - C17H23N2O2
m/z 315
C19H27N2O2
M + H

2. **Compound 2 (317.2882):**

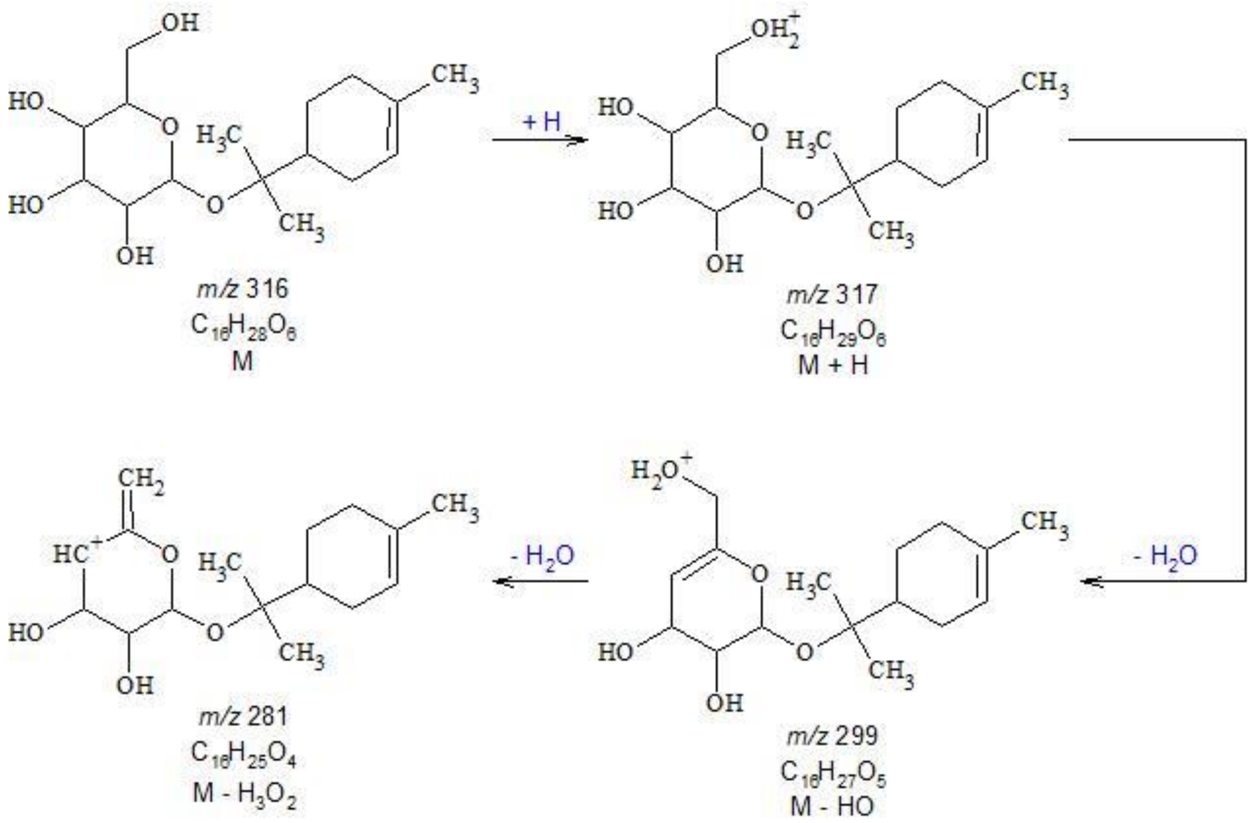

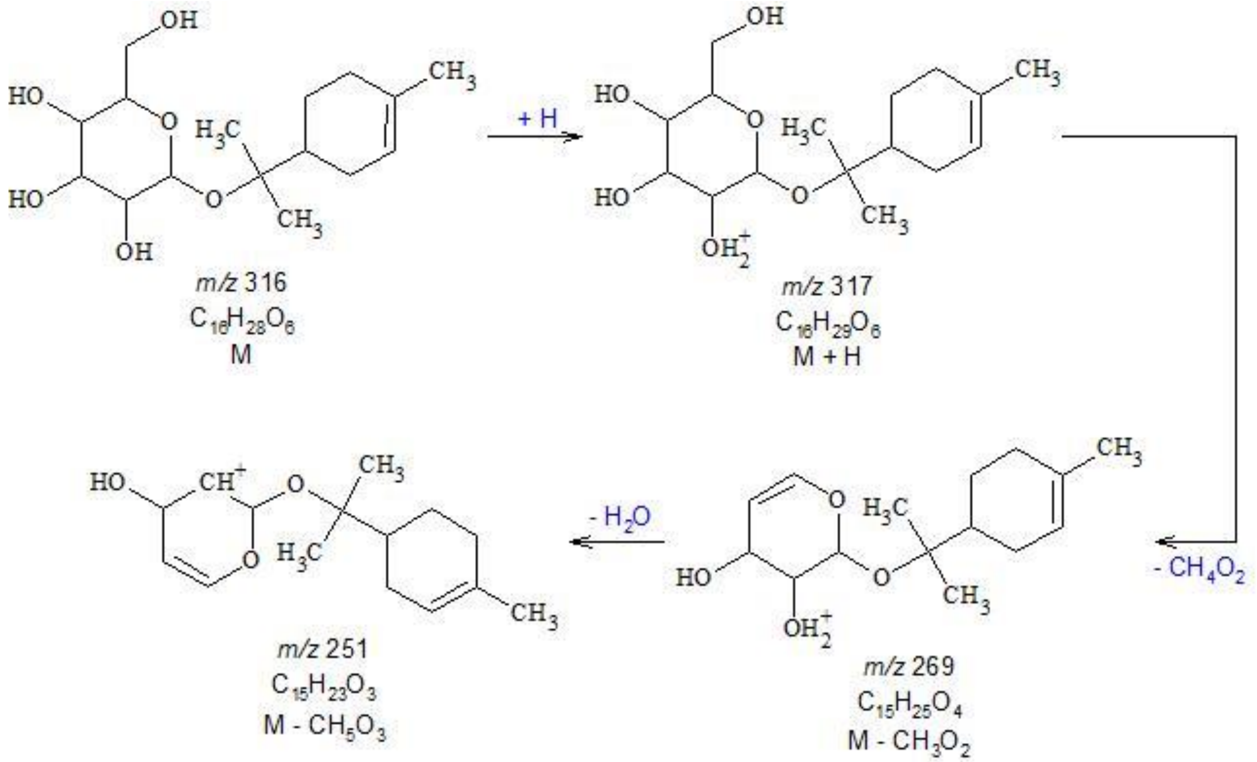
m/z 316
C16H28O6
M
+ H
m/z 317
C16H29O6
M + H
- CH4O2
m/z 269
C15H25O4
M - CH3O2
- H2O
m/z 251
C15H23O3
M - CH5O3

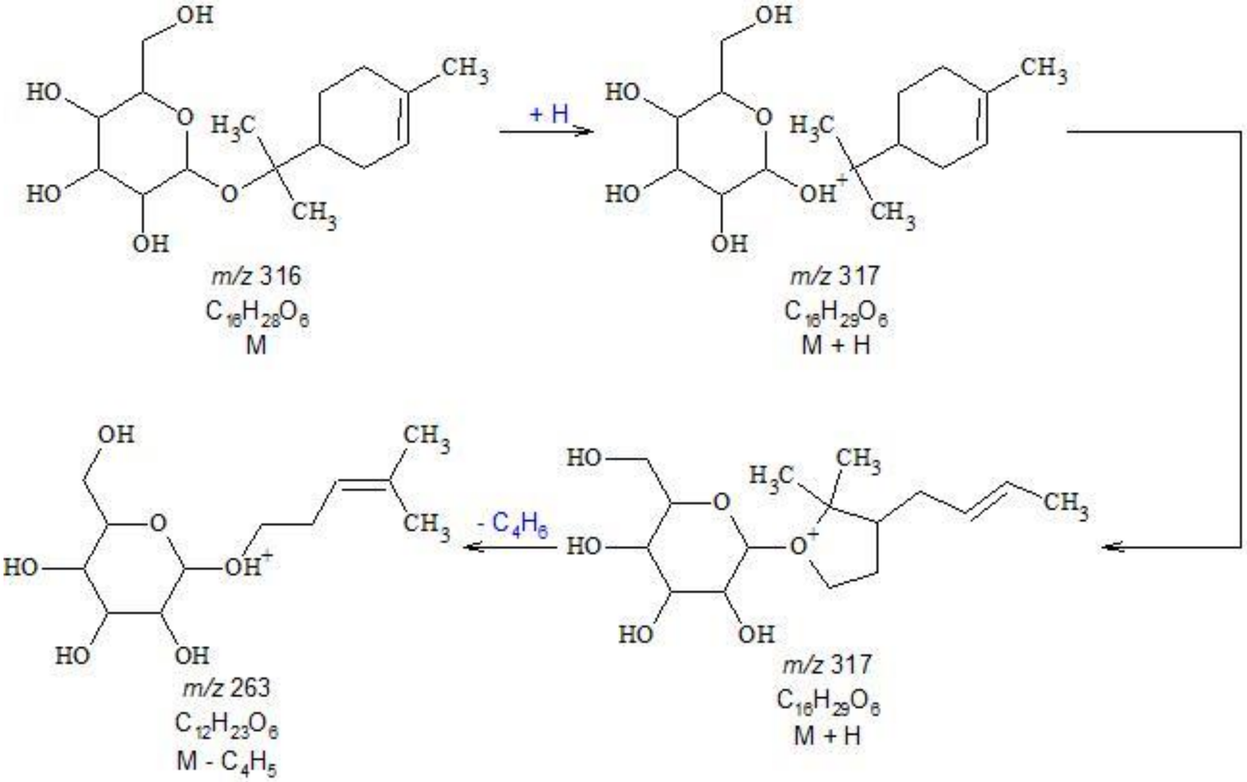
OH
HO
O
HO
OH
$H_3C$
O
$CH_3$
$CH_3$
m/z 316
$C_{16}H_{28}O_6$
M
+ H
m/z 317
$C_{16}H_{29}O_6$
M + H
$OH^+$
HO
$O^+$
$H_3C$
$CH_3$
$CH_3$
$CH_3$
- $C_4H_6$
$OH^+$
m/z 263
$C_{12}H_{23}O_6$
M - $C_4H_5$

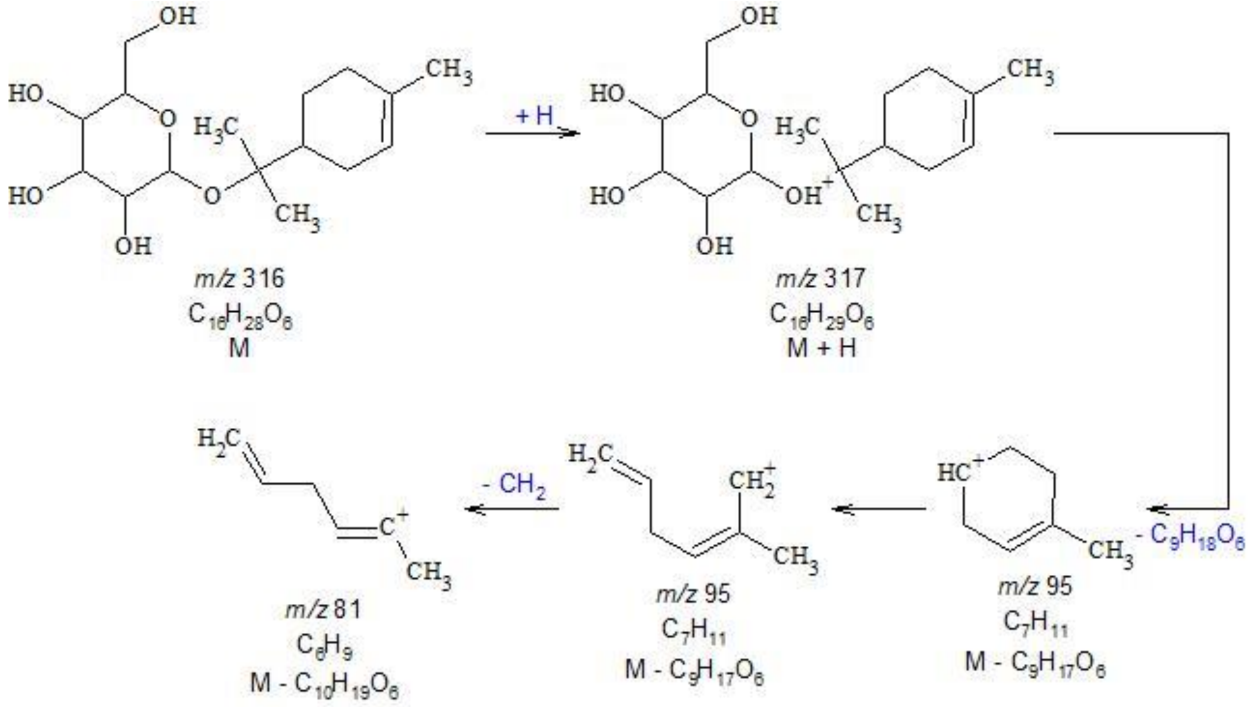
+ H
m/z 316
C16H28O6
M
m/z 317
C16H29O6
M + H
- C9H18O6
m/z 95
C7H11
M - C9H17O6
m/z 95
C7H11
M - C9H17O6
- CH2
m/z 81
C6H9
M - C10H19O6

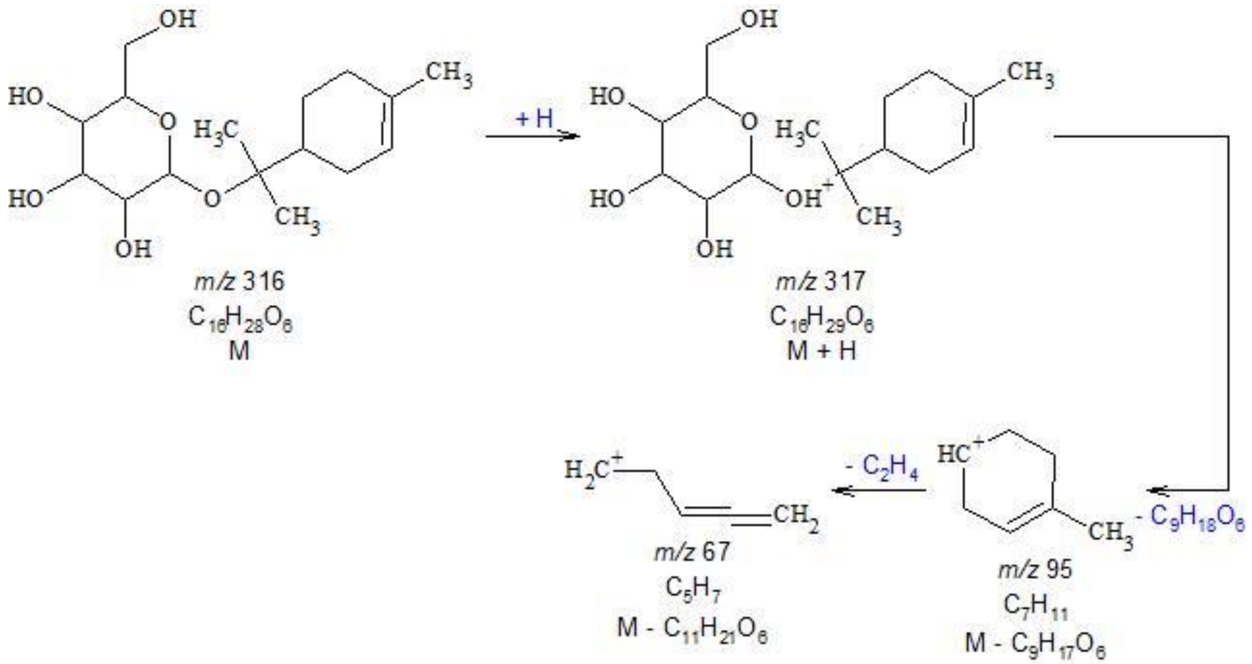
+ H
m/z 316
C16H28O6
M
m/z 317
C16H29O6
M + H
- C9H18O6
m/z 95
C7H11
M - C9H17O6
- C2H4
m/z 67
C5H7
M - C11H21O6

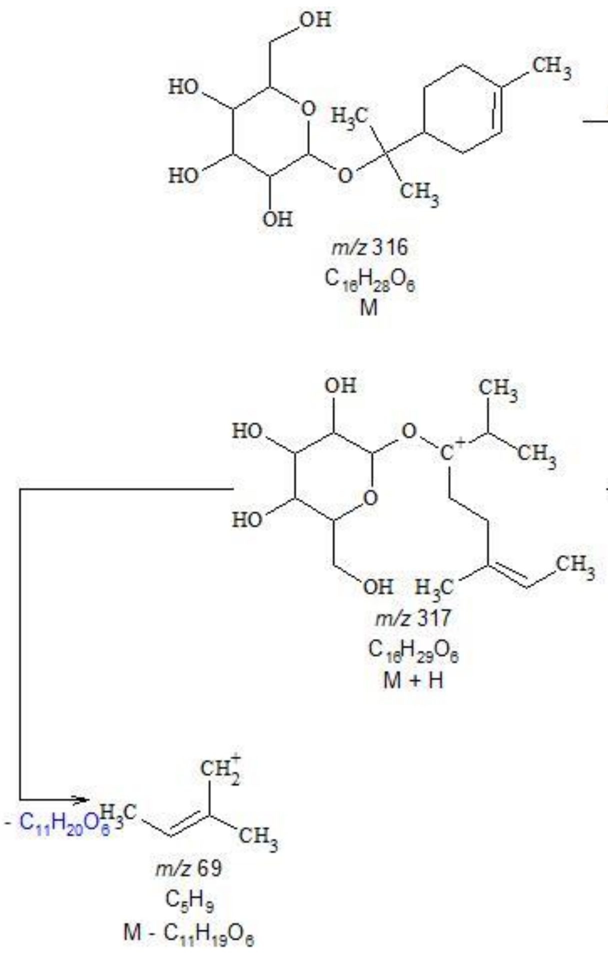

m/z 316
C16H28O6
M
+ H
m/z 317
C16H29O6
M + H
m/z 317
C16H29O6
M + H
m/z 317
C16H29O6
M + H
- C11H20O6
m/z 69
C5H9
M - C11H19O6

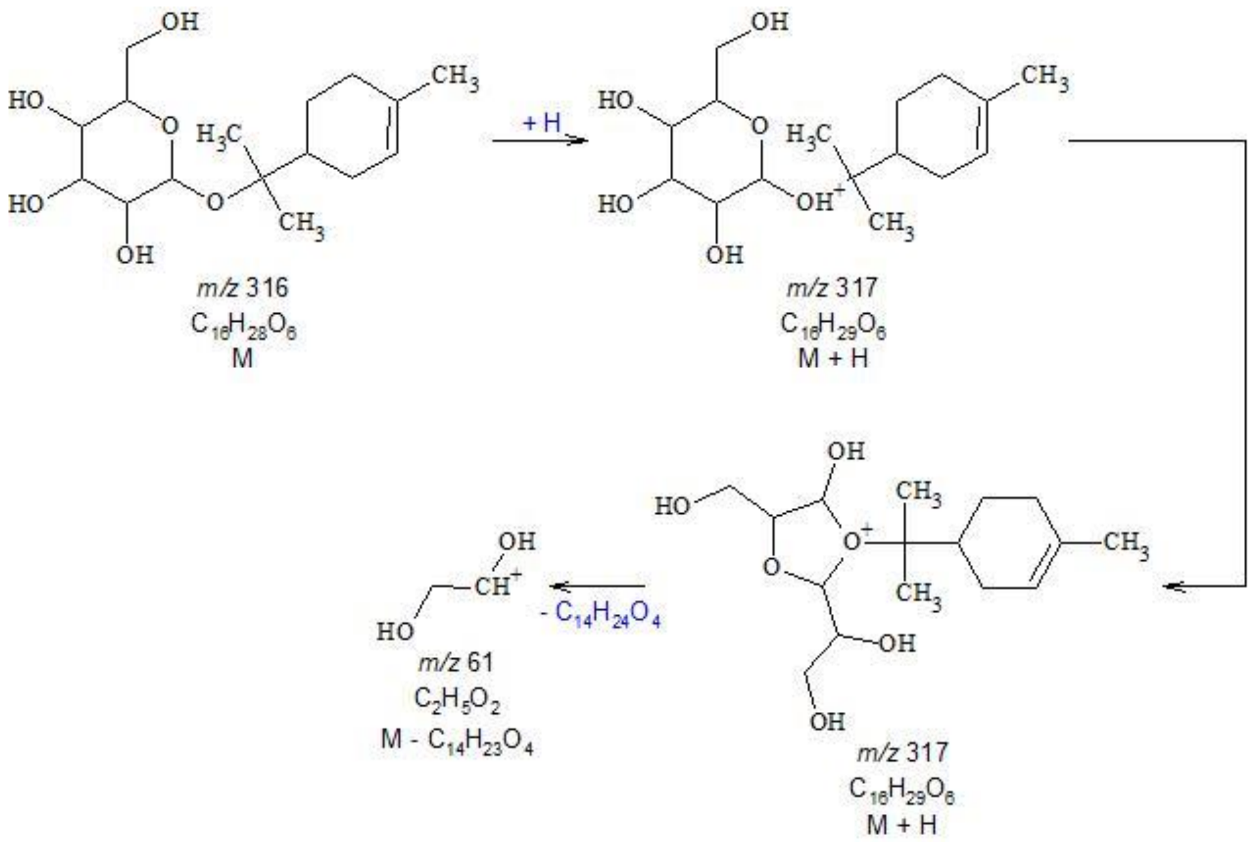

m/z 316
C16H28O6
M
+ H
m/z 317
C16H29O6
M + H
m/z 317
C16H29O6
M + H
- C14H24O4
m/z 61
C2H5O2
M - C14H23O4

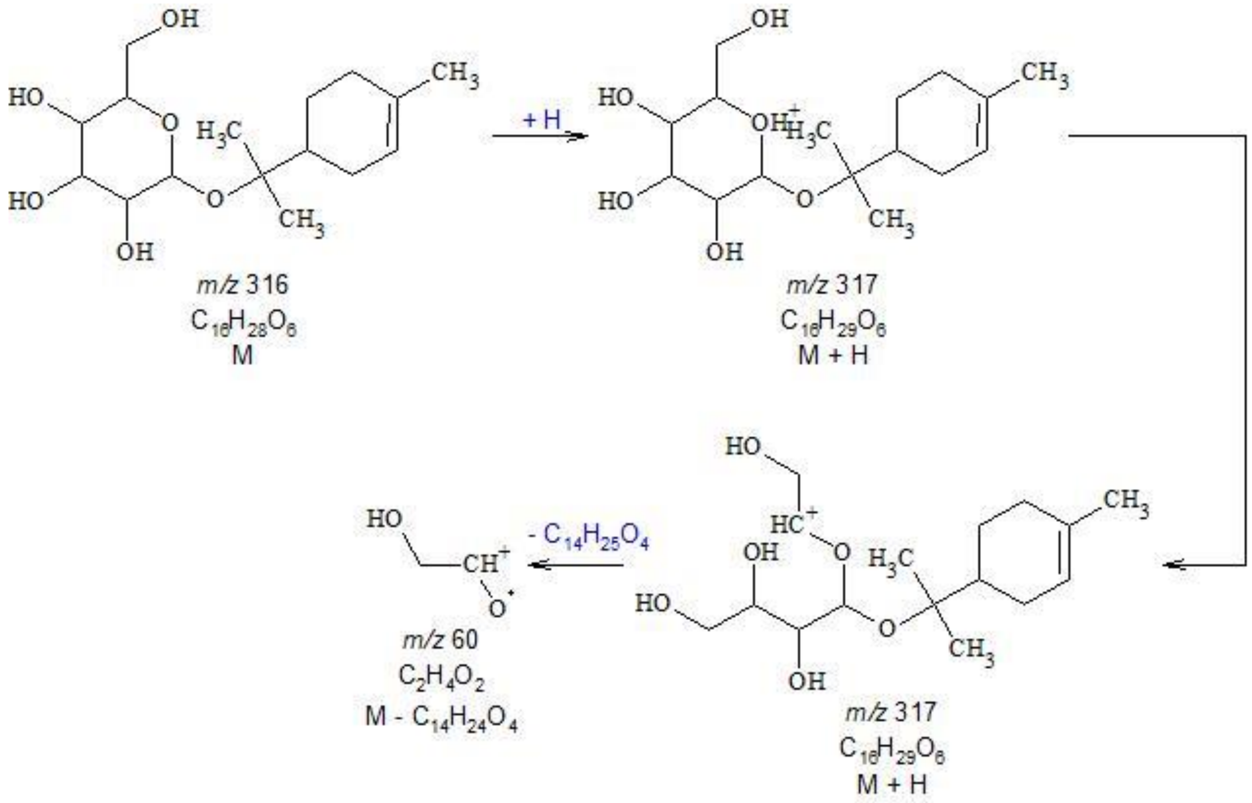

OH
HO
O
H3C
CH3
HO
OH
CH3
m/z 316
C16H28O6
M
+ H
OH+
m/z 317
C16H29O6
M + H
HO
HC+
- C14H25O4
CH+
O+
m/z 60
C2H4O2
M - C14H24O4
m/z 317
C16H29O6
M + H

3. **Compound 3 (349.2127):**

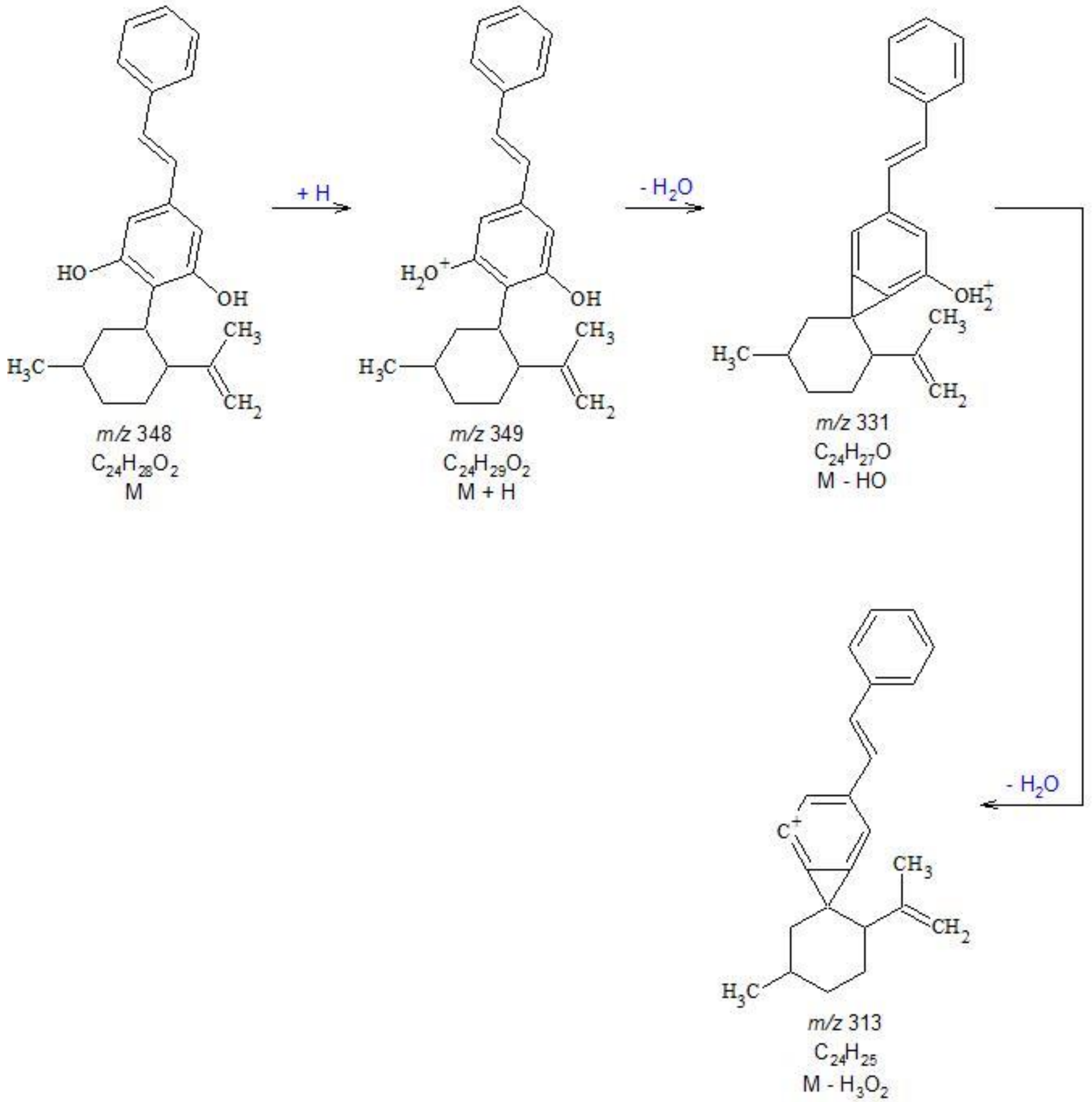

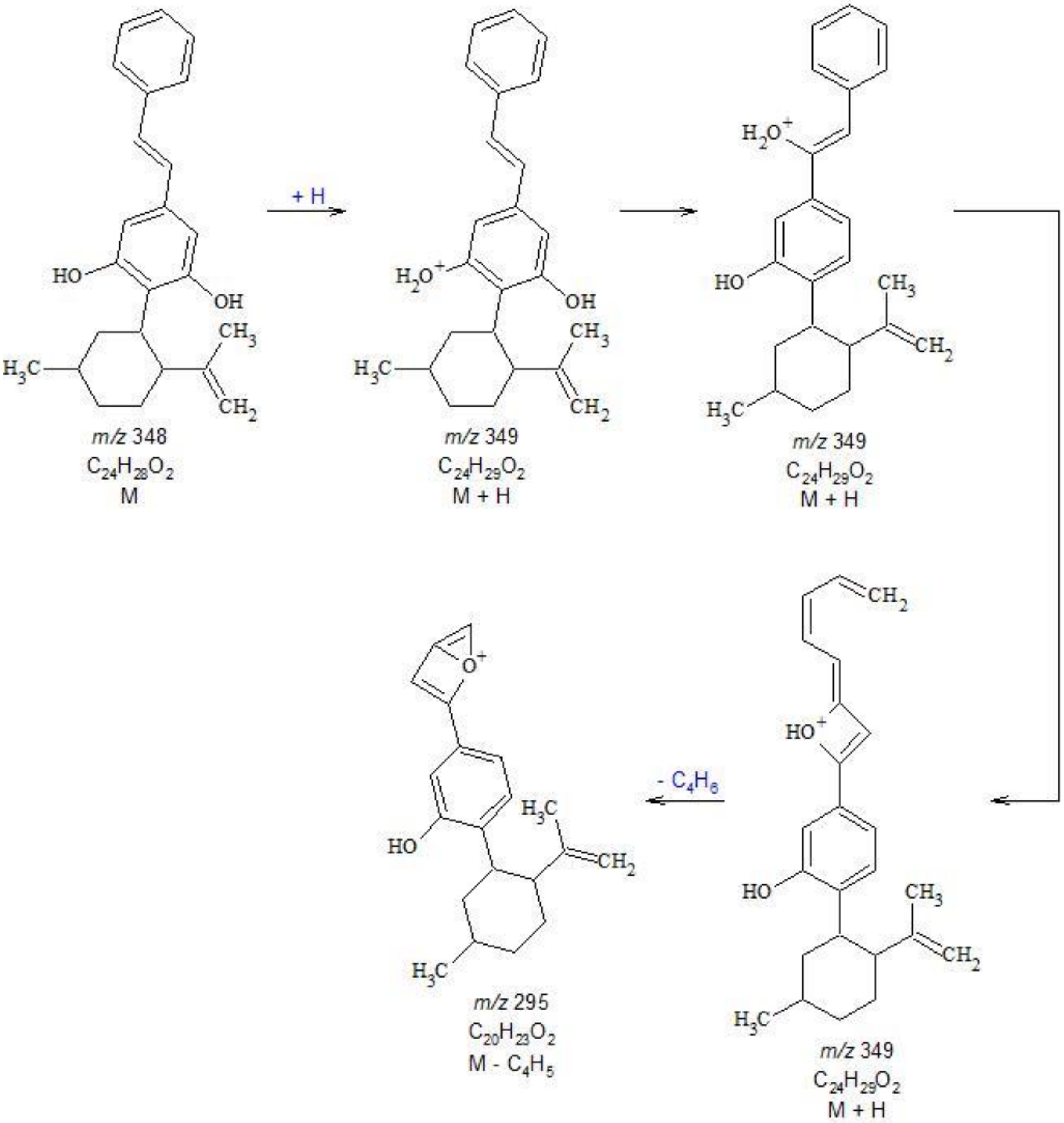
+ H
m/z 348
C24H28O2
M
m/z 349
C24H29O2
M + H
m/z 349
C24H29O2
M + H
- C4H6
m/z 349
C24H29O2
M + H
m/z 295
C20H23O2
M - C4H5

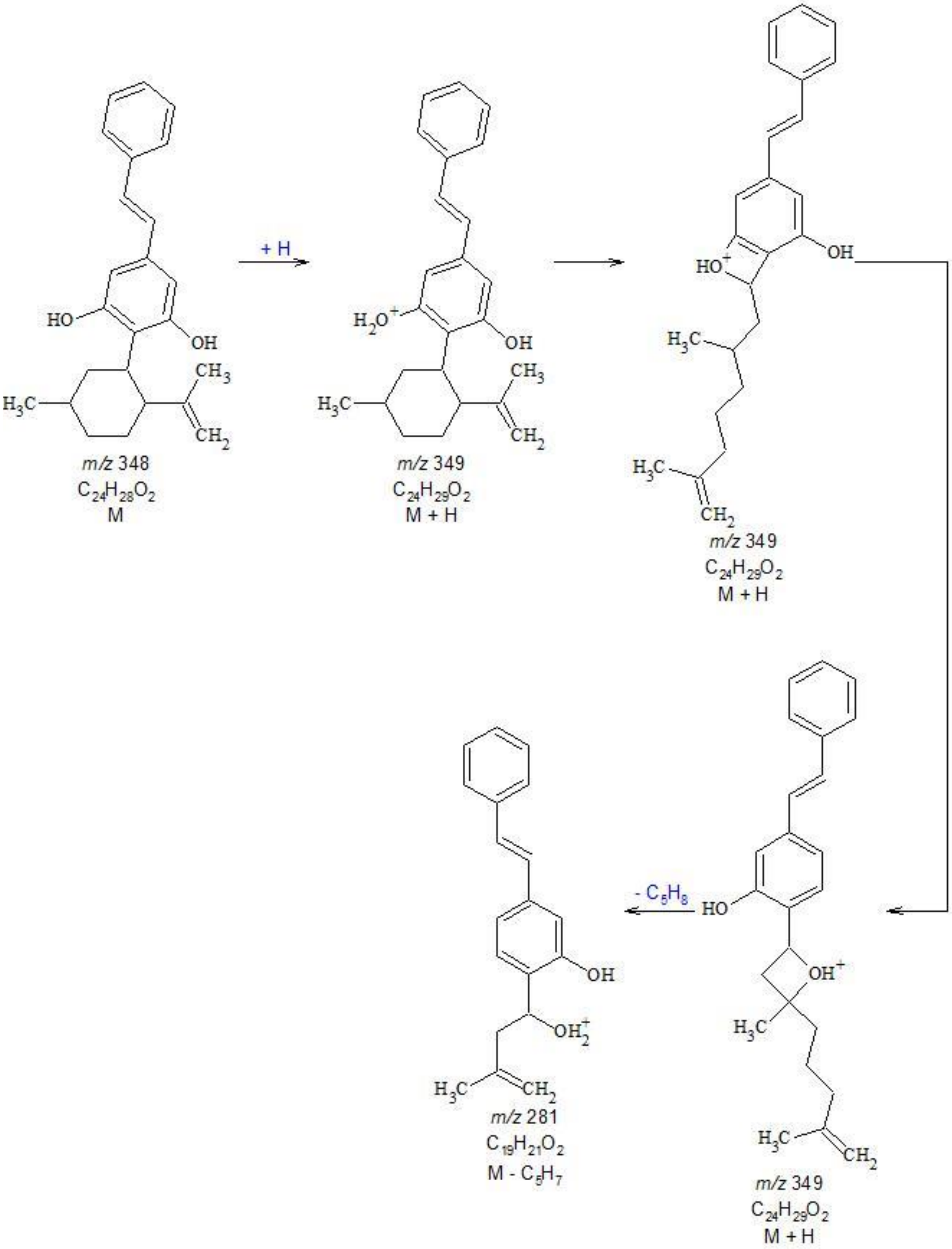

HO
OH
CH3
H3C
CH2
m/z 348
C24H28O2
M
+ H
H2O+
OH
CH3
H3C
CH2
m/z 349
C24H29O2
M + H
HO+
OH
H3C
H3C
CH2
m/z 349
C24H29O2
M + H
HO
OH+
H3C
H3C
CH2
m/z 349
C24H29O2
M + H
- C5H8
OH
OH2+
H3C
CH2
m/z 281
C19H21O2
M - C5H7

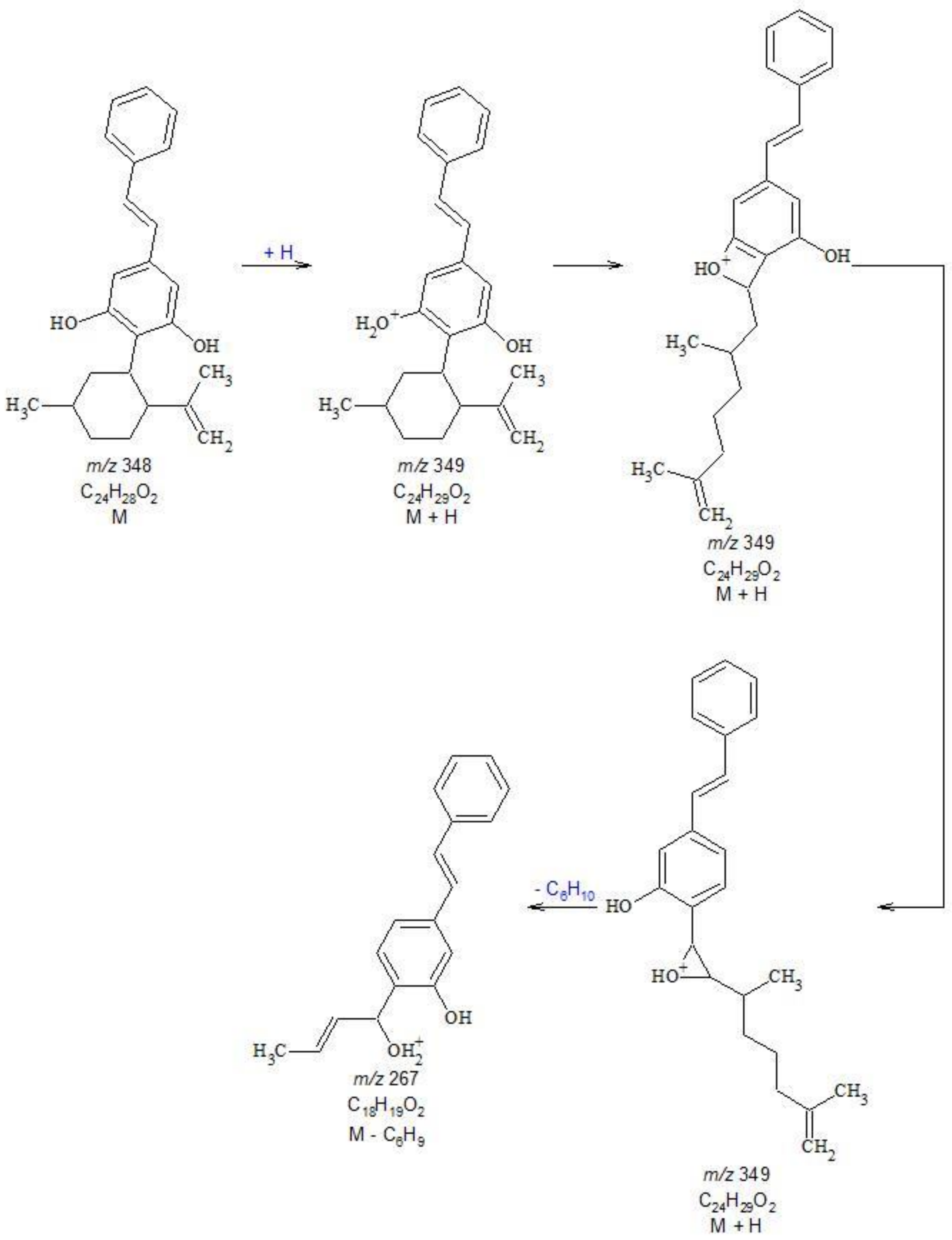

+ H
HO
OH
CH3
H3C
CH2
m/z 348
C24H28O2
M
H2O+
OH
CH3
H3C
CH2
m/z 349
C24H29O2
M + H
HO+
OH
H3C
H3C
CH2
m/z 349
C24H29O2
M + H
- C6H10
HO
HO+
CH3
CH3
CH2
m/z 349
C24H29O2
M + H
H3C
OH2+
OH
m/z 267
C18H19O2
M - C6H9

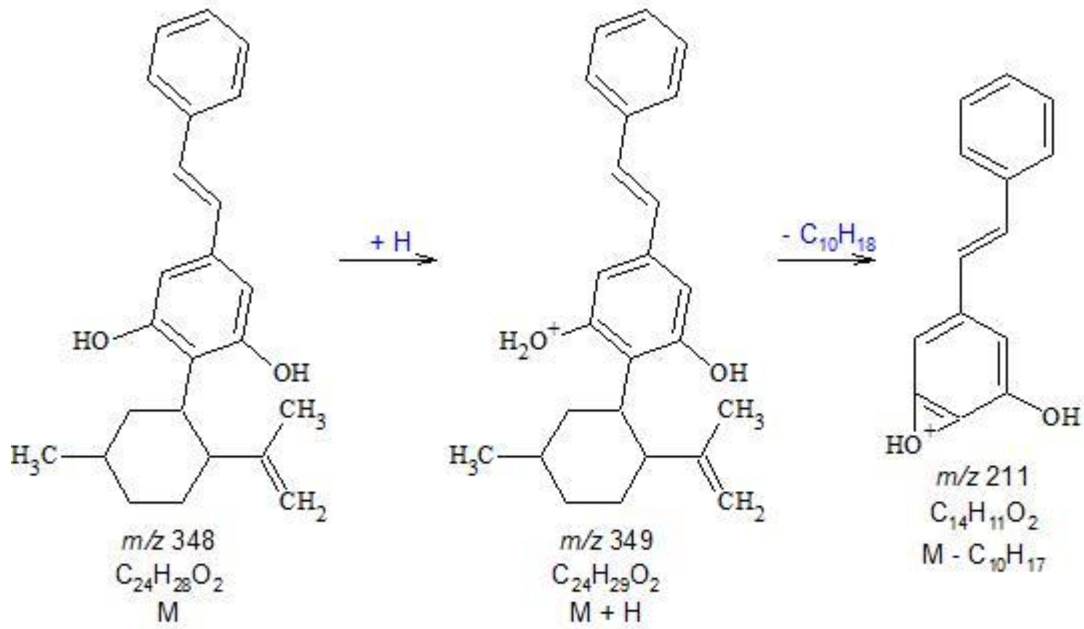

+ H
- C10H18
HO
OH
H3C
CH3
CH2
H2O+
HO+
m/z 348
C24H28O2
M
m/z 349
C24H29O2
M + H
m/z 211
C14H11O2
M - C10H17

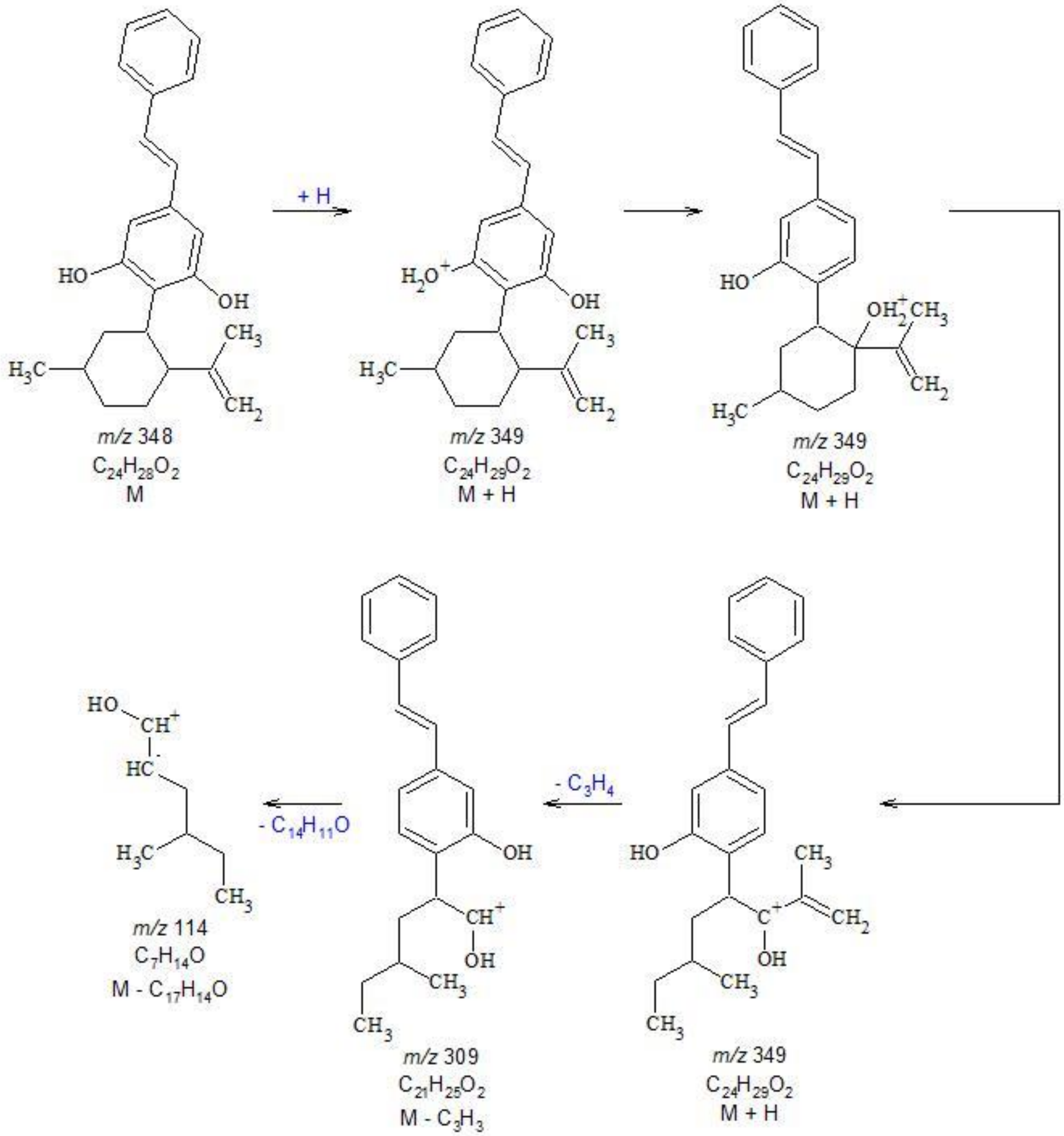
+ H
m/z 348
C24H28O2
M
m/z 349
C24H29O2
M + H
m/z 349
C24H29O2
M + H
- C3H4
- C14H11O
m/z 349
C24H29O2
M + H
m/z 309
C21H25O2
M - C3H3
m/z 114
C7H14O
M - C17H14O
